# An Infusible Decellularized Extracellular Matrix Material Binds to Vasculature in Infarcted Myocardium and Induces Pro‐Reparative Gene Expression Following Acute Myocardial Infarction Through Inherent Avidity and Bioactive Signaling

**DOI:** 10.1002/advs.78446

**Published:** 2026-10-29

**Authors:** Michael B. Nguyen, Alexander Chen, Quincy Lyons, Van K. Ninh, Maxwell C. McCabe, Colin Luo, Benjamin D. Bridgelal, Connor Uhre, Julian Cheng, Kate E. Reimold, Selena Cao, Kirk C. Hansen, Kevin R. King, Karen L. Christman

**Affiliations:** ^1^ Shu Chien‐Gene Lay Department of Bioengineering University of California San Diego La Jolla California USA; ^2^ Sanford Consortium for Regenerative Medicine University of California San Diego La Jolla California USA; ^3^ Program in Materials Science and Engineering University of California San Diego La Jolla California USA; ^4^ Department of Medicine Division of Cardiology University of California San Diego La Jolla California USA; ^5^ Department of Biochemistry and Molecular Genetics School of Medicine University of Colorado Aurora Colorado USA; ^6^ Sanford Stem Cell Institute University of California San Diego La Jolla California USA

**Keywords:** avidity, basal lamina, biomaterial, cell biology, decellularization, endothelial stem cell, extracellular matrix, gene expression, in vivo, vascular permeability

## Abstract

To treat acute myocardial infarction (MI), we previously engineered an infusible decellularized extracellular matrix (iECM) biomaterial that reduces vascular permeability and exerts immunomodulatory and pro‐reparative effects. To probe the heterogenous contents of iECM and its contribution to infarct localization and downstream biological function, we separated iECM into high and low molecular weight (MW) components. Though mass spectrometry showed similar compositions, biochemical analysis and transmission electron microscopy revealed higher sGAG content within the nanofibrillar high MW, while the globular low MW had more exposed functional groups. These differences translated to a higher binding efficiency of the high MW to ECM proteins and cell surface receptors under flow, while the low MW drove the reduction in vascular permeability. Neither component alone achieved the infarct retention levels of complete iECM, showing that the two components exhibit dependent infarct localization and both are necessary for iECM's overall function. Via single‑nucleus RNA sequencing, both components elicited comparable immunomodulatory and pro‑reparative transcriptional profiles, suggesting that the similarity in composition likely drives the transcriptional response. These findings elucidate the material properties of an intravascularly deliverable ECM biomaterial and how those properties relate to downstream biological behavior, enabling future work investigating nanomaterial mimicry and improvements in material processing and potency.

## Graphical abstract

To better characterize an infusible extracellular matrix (iECM) material, we applied size‐based fractionation to separate out components into two major fractions. We characterized these fractions to understand how material properties such as composition, morphology, and biochemical properties affect bioactive effects in the context of acute myocardial infarction, providing insight into further development of infusible, targeting biomaterials.

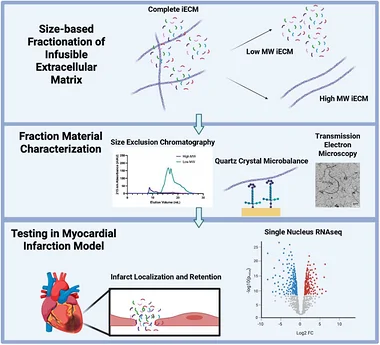

## 1 Introduction

Ischemic heart diseases continue to be a significant health care burden worldwide, with an estimated 9 million deaths attributed to ischemic heart disease [1]. A major contributor to ischemic heart disease is acute myocardial infarction (AMI), which is characterized by the restriction of blood flow to the myocardium, often caused by the buildup of atherosclerotic plaque in the coronary arteries. This ischemic damage is characterized by cardiomyocyte death, endothelial dysfunction, increased vascular permeability, immune cell infiltration, and oxidative stress [2, 3]. As a result of injury, cardiac function is impaired, and the resulting compensatory adaptations in the weeks and months following AMI is known as negative left ventricular (LV) remodeling, which ultimately leads to heart failure [4, 5]. The most common treatment for AMI is percutaneous coronary intervention, which opens the artery via balloon angioplasty [1]. This reperfusion is necessary to save the at‐risk tissue; however, the reintroduction of oxygen rich blood further exacerbates the inflammation and is known as ischemia‐reperfusion injury [6, 7].

For restoring cardiac function after MI, biomaterial treatments such as cardiac patches and hydrogels have been utilized to exert immunomodulatory effects and support tissue repair [8]. While cardiac patches have shown therapeutic effects in preclinical models [9], they require invasive surgery to access the epicardium for implantation, bringing with it significant risk of complications. Similarly, the injection of biomaterials into the myocardium can mitigate inflammation and support cardiac repair [10, 11, 12, 13] but will be limited to sub‐acute and chronic delivery timepoints clinically, as injection into the weakened myocardium at acute timepoints carries the risk of arrythmias and ventricular rupture [14, 15, 16]. As such, to treat MI and ischemia‐reperfusion injury acutely, infusible biomaterials that are intravascularly delivered have seen increased popularity [17]. However, these materials require a method of localization to the injured myocardium to minimize off‐target effects and improve treatment efficacy.

For many applications, decellularized extracellular matrix (ECM) biomaterials have seen great interest due to their inherent immunomodulatory and pro‐reparative motifs derived from their source tissue [18], as well as their cost‐effective production. To leverage these properties for reducing AMI induced negative LV remodeling in a minimally invasive manner, we recently developed an intravascularly infusible ECM material (iECM) [19]. Through further processing of pepsin digested ECM (Figure 1a), iECM can be delivered immediately upon reperfusion without thrombus formation despite containing collagen‐like fibril components [19]. iECM localizes to vasculature in areas of inflammation, reduces vascular permeability and encourages vascular healing, exerts immunomodulatory effects, and improves cardiac function in both small and large animal models of AMI [19]. It also reduced inflammation and improved animal survival in a model of severe systemic inflammation [20].

**FIGURE 1:**
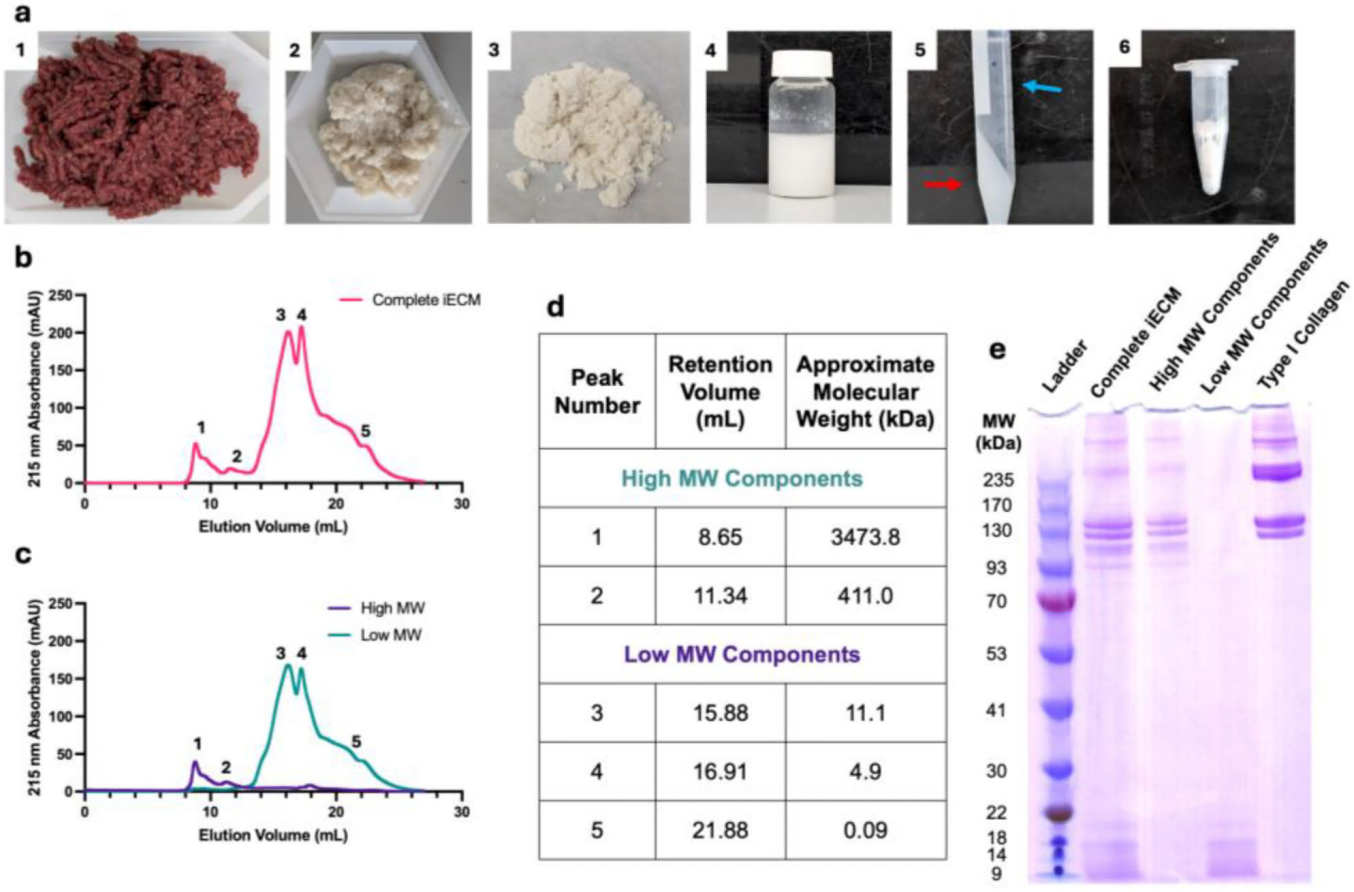
Characterization of iECM molecular weight distribution and separation of iECM components into two fractions. a) Fabrication of iECM by 1) mincing porcine LV tissue, 2) decellularizing with SDS, 3) lyophilizing and milling into a powder, 4) partially digesting with pepsin, 5) centrifuging to pellet out large particulate matter (red arrow), 6) sterile filtering the supernatant (blue arrow) and lyophilizing for storage. SEC traces of b) complete iECM and c) iECM separated into High MW components (blue) and Low MW components (purple). d) Quantification from SEC traces showing approximate molecular weight of iECM components. d) SDS‐PAGE of complete iECM, High MW components, Low MW components, and collagen type I demonstrating efficient separation of iECM into two components.

While many studies have been done to determine how ECM materials work in the context of solid and hydrogel scaffolds, the mechanisms through which intravascularly delivered ECM materials interact with vasculature in inflamed tissue and exert therapeutic effects have yet to be understood. For synthetic nanomaterials, it has often be hypothesized that targeting of inflamed vasculature can be achieved through the use of anisotropic materials [21, 22] that more effectively marginate within the vasculature [23]. However, these materials often use a targeting moiety to bind to only a single component, such as upregulated endothelial cell receptors that occur with inflammation, like intracellular adhesion molecule 1 (ICAM) [24], or the exposed underlying type IV collagen in the basement membrane in injured blood vessels [25]. Despite these rational targets, there has been no in vivo histological evidence showing these materials binding to the gaps in permeable endothelium. Given iECM was shown to have this unique localization [19], we sought to investigate how iECM targets, which could inform the design of new nanomaterial therapeutics, as well as investigate the fundamental biochemical and biological behaviors of the material as part of our development of an efficacious minimally invasive treatment for AMI.

In this study, we applied size‐based fractionation to separate iECM into its main components, enabling us to study how each component behaves in the context of inflammation and AMI. This method yielded two major components based on apparent molecular weight. We first characterized the morphological and biochemical properties to understand how each component would behave once delivered intravascularly. We then investigated the role of these components in reducing vascular permeability and inducing repair and regeneration of cardiac tissue via next generation sequencing. Altogether, this manuscript provides evidence for a new avenue for targeting and treating diseases associated with acute inflammation, leveraging both heterogeneous shape and specific high avidity binding as a mechanism for targeting and treating permeable vasculature.

## 2 Results and Discussion

### 2.1 iECM Consists of a High Molecular Weight Nanofibrillar Component and a Soluble, Low Molecular Weight Peptide Component

iECM was fabricated using decellularized porcine LV myocardial tissue similar to previous reports [19] (Figure 1a). The degree of decellularization was measured using a double‐stranded DNA (dsDNA) assay, showing less than 5 ng dsDNA per mg of ECM as well as sufficient sodium dodecyl sulfate (SDS) removal (Figure S1a,b) [26]. Via SDS polyacrylamide gel electrophoresis (SDS‐PAGE), we saw that the banding pattern of iECM was qualitatively consistent with previous batches made by others using the same protocol (Figure 1e) [19, 27, 28, 29]. Overall, this suggested that the iECM fabrication process is robust and reproducible.

To quantify the relative composition of iECM based on apparent molecular size, we analyzed iECM with size exclusion chromatography (SEC) (Figure 1b). The processing of iECM with 0.22 µm filtration and a viscosity comparable to saline [19] enabled SEC analysis of the entirety of iECM, in contrast with hydrogel formulations of decellularized ECM [30]. The mobile phase was phosphate buffered saline (PBS) to simulate the salt conditions that iECM would experience after intravascular delivery. We found that iECM followed a bimodal distribution comprised of a high molecular weight (High MW) component (apparent MW > 100 kDa) and a low molecular weight component (Low MW) (apparent MW < 10 kDa) (Figure 1b–d). Based on 215 nm absorbance [31], high MW components made up approximately 15% of the total iECM composition, while the low MW components constituted the remaining 85%. Although peaks on the ends of the SEC trace (peaks 1 and 5) fall outside of the reported separation range of the SEC column used, this method still allows us to quantify the relative composition of iECM based on their apparent molecular weight because of the clear separation of the high and low MW components as determined by SEC.

To separate out the two components based on MW, we leveraged the differences in solubility. The low MW components could be dissolved using 50% ethanol in water, while the high MW components precipitated out (Figure S1c). After separation and weighing, the percentages of each component was found to be approximately 19% and 81% high MW and low MW by mass, with this difference due to differences in the amino acid composition [31] and the presence of non‐protein components in the high MW component that did not appear on SEC. Separation of iECM components was confirmed via SEC and SDS‐PAGE, where the peaks and bands of iECM components matched well to complete iECM, confirming that the conformation of the components was not affected by the separation process (Figure 1c,e). Overall, these data show that the complete iECM can be efficiently fractioned into its 2 predominant components and studied independently.

To investigate the composition, label‐free quantification mass spectrometry was performed (Tables S1 and S2) to show the abundances of identified protein categories that the two components are derived, based on mean measured signal intensity (Figure 2a). While no category was statistically significant, the data suggest the HMW does have less non‐fibrillar collagen‐derived peptides compared to the LMW. This was corroborated by negatively stained transmission electron microscopy (nsTEM), which revealed that the high MW component contained fragmented, flexible nanofibers (Figure 2b), similar to what was previously seen with complete iECM [19], and the low MW component contained globular peptides (Figure 2c). To further probe differences in the composition, we compared the overlap in the number of identified parent ECM proteins without factoring in relative abundances to identify any unique ECM features in either of the two components. Overall, 94% ± 3.5% of the identified parent ECM proteins in the high MW were found in the low MW and 91% ± 0.1% of the identified ECM proteins in the low MW were found in the high MW. For peptide sequences, 71% ± 2.3% of peptide sequences identified in the high MW were found in the low MW and 76% ± 0.6% of peptide sequences identified in the low MW were found in the high MW. This showed that both components were derived from the same parent ECMs and that peptide fragments were similar, although not identical. The primary difference between the two components is the relative abundance of those same digested ECM proteins. This is likely due to the nanofibrils, which are comprised of numerous fibrillar collagen monomers, and would result in a greater abundance of the fibrillar collagen peptides identified after mass spectrometry sample preparation. Additionally, iECM is pepsin digested during its fabrication process and the high MW and low MW components likely represent digested ECM at different stages of the digestion process. Therefore, while the two components have the same parent ECMs, a greater abundance of biochemical attributes is exposed within the more digested, globular peptides of the low MW as compared to the more intact nanofibrils of the high MW component. It is however important to note when considering this mass spectrometry data that high MW and low MW were both pepsin digested as part of material processing and then trypsin digested for the typical mass spectrometry protocol needed for ECM, which then identifies peptides based on databases for tryptic fragments. The pepsin digestion will therefore skew the identification of sequences and lead to more unidentified sequences. Thus, true compositional analysis with these pepsin‐digested complex ECM biomaterials is challenging.

**FIGURE 2:**
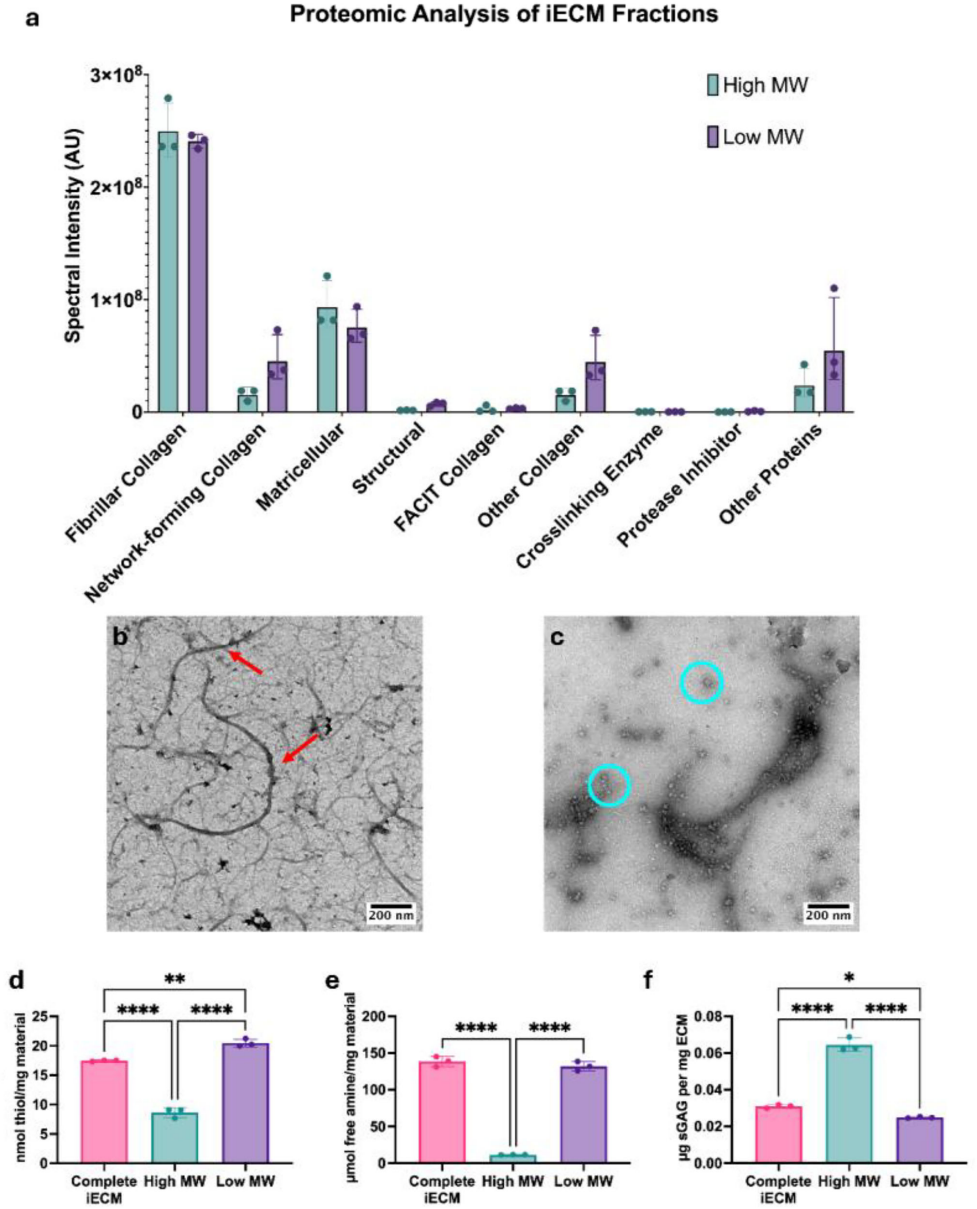
Compositional, morphological, and biochemical analysis of iECM and its components. a) The fractions were analyzed via label‐free quantification of trypsin‐digested mass spectrometry for original protein content, with the spectral intensity shown for the High MW components and Low MW components. The morphologies were also visualized via TEM for morphology: b) high MW and c) low MW at 30kx magnification. Biochemical assays measuring the d) thiol content, e) free amine content, and f) sGAG content of iECM, low MW, and high MW components. Red arrows denote nanofibrils and cyan circles denote globular and nanofibril peptides. Statistical significance was determined by one‐way ANOVA with Tukey's post‐hoc test (*n* = 3 technical replicates). Error bars represent standard deviation.^*^ *p* < 0.05, ^**^ *p* < 0.01, ^***^ *p* < 0.001, ^****^ *p* < 0.0001.

We next characterized the biochemical differences between the components. The free thiol component was significantly greater within the low MW component (Figure 2d). Previous findings determined that iECM was capable of acting as an antioxidant by sequestering reactive oxygen species with one of the mechanisms via free thiol content [12, 19]. Seeing the higher thiol content in the low MW with suggests the low MW portion of iECM was responsible for that previously observed antioxidative behavior [12, 19]. We also found that the low MW component contained significantly more free amines (Figure 2e) compared to the high MW component. This was expected given the hypothesis that the low MW was comprised predominantly of digested ECM peptides, as the cleaving of ECM proteins into peptides would generate more free amines through the breakage of amide bonds. Finally, we found that the high MW component contained most of the sulfated glycosaminoglycans (sGAG) (Figure 2f). sGAGs, typically found as part of proteoglycans, are desirable within decellularized ECM materials for their bioactivity [26]. Therefore, based on the biochemical readouts, both components may contribute a unique mechanism to the overall biological activity of the complete iECM.

### 2.2 iECM and its Components Specifically Bind to Extracellular Matrix Proteins and Cell Surface Receptors

We next investigated how these observed differences translated to the binding capabilities of iECM components. Since iECM was found to colocalize with permeable vasculature post‐MI [19], we evaluated binding to underlying ECM components exposed with permeable vasculature, such as laminins [32, 33], type IV collagen [32, 33], and type I collagen [34]. Additionally, endothelial cell damage and dysfunction caused by MI result in upregulated integrin α1β1 [35, 36], ICAM [37], and P‐selectin [38]. Quartz crystal microbalance (QCM) is sensitive to surface binding under flow and has previously been used to evaluate peptide binding to ECM surfaces [39] and antibodies to laminin surfaces [40]. Since the moles of the iECM components cannot be determined due to the polydispersity, we evaluated relative binding efficiency for a target by delivering the high and low MW at their respective proportions in complete iECM. Using previous protocols [41], ECM proteins and cell receptor proteins were conjugated to functionalized lipids. Since the lipid‐protein coating had a high dissipation, it was viscoelastic and thick. Thus, the lower overtones more accurately represent the binding to the conjugated proteins at the top of the thick layer [39]. We reported the third overtone frequency shift as previously done for hydrated laminin [40] and other QCM results [42, 43, 44]. Using antibodies to validate target coating, as was investigated for laminin [40], we found varying degrees of antibody binding (Figure S2a) due to the variation in the efficiency of ECM and cell receptor protein conjugation. Nonetheless, our antibody binding to laminin was similar to previous results [40], giving confidence in successful conjugation.

Overall, the high MW component had increased binding to all of the coatings except for type IV collagen and P‐selectin (Figure 3a,b). This suggested that the high MW had the greatest apparent specificity to the various components exposed in the permeable vasculature. In relation to the physical structure, it was found that anisotropic nanomaterials, like nanofibers, more effectively marginate and exhibit multivalent binding under flow [21]. From the TEM images, the high MW nanofibrils show curved, flexible morphologies suggesting a more flexible structure, which has also been shown to improve binding [21]. The combination of flexibility and nanofibriliar structure may enable multivalent binding to ECM proteins and cell surface receptors, improving binding under dynamic flow. Altogether, these data show that iECM and its components have specific binding to multiple targets within the exposed ECM and cellular components of inflamed leaky vasculature.

**FIGURE 3:**
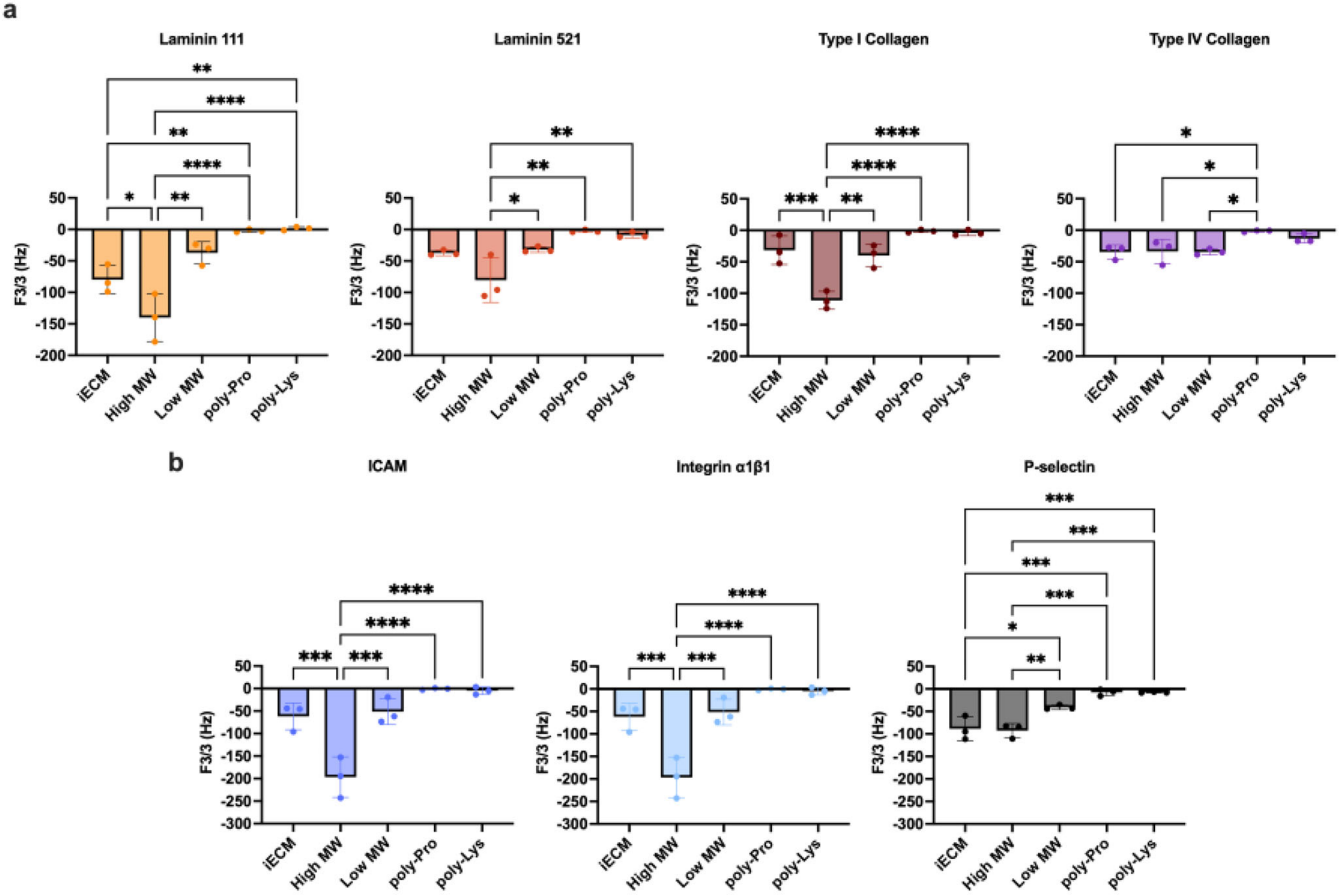
Evaluation of binding of iECM and its components to immobilized ECM proteins and cell receptors via quartz crystal microbalance. Display of the average third harmonic frequency shift values with standard deviations to show variance for binding to a) ECM components and b) cell surface receptors. Lower frequency represents greater binding of the flowed iECM or components to the immobilized protein of interest. Comparisons were only made between the same chemical conjugated coating. Statistical significance determined One‐way ANOVA with Tukey's post‐hoc test (*n* = 3 technical replicates). Error bars represent standard deviation. ^*^ *p* < 0.05, ^**^ *p* < 0.01, ^***^ *p* < 0.001, ^****^ *p* < 0.0001.

Interestingly, the high MW binding for laminin 111, type I collagen, ICAM, and integrin α1β1 was significantly higher than both complete iECM and low MW. This suggested interactions between the components within the flow of complete iECM, therefore allowing the high MW to bind more efficiently when on its own. To assess the interactions of the components, we coated the surface with the high MW or low MW component and flowed the opposite component (Figure S2b). The difference in binding was likely because the low MW had significantly more free amines (Figure 2f) and therefore increased conjugation efficiency to the lipid surface, allowing for a greater degree of binding sites. However, this finding primarily highlights the interaction between the two components and explains why complete iECM binding is not a sum of high MW and low MW. Instead, the high and low MW components likely interact with each other under flow and prevent higher‐efficiency binding to the surface as compared to high MW alone. Additionally, these data suggest that the interaction between the two components may impact the iECM localization in vivo.

As controls, poly‐L‐proline (neutral) and poly‐L‐lysine (positive) with similar MWs to the low MW component were used. The poly‐L‐lysine had a larger shift in frequency compared to the poly‐L‐proline suggesting an improvement of binding with an electrostatic charge. However, relative to the iECM and its components, the frequency shifts were significantly lower for most targets (Figure 3). This suggested that complete iECM and its components have specific binding to the various ECM and cellular receptors, therefore confirming the previously observed colocalization with the gaps of permeable vasculature as specific binding. This is not surprising given that self‐ and cross‐ binding between ECM proteins is well known, both in the context of native tissue and solid biomaterial development [45, 46]. Our data demonstrate that the distinct components of iECM retain the unique binding behavior to both ECM proteins and cell surface receptors while under flow, enabling them to target multiple aspects of endothelial dysfunction. The findings highlight a unique behavior of iECM with varying binding affinities to other ECM components and cellular receptors and illustrate the advantage of diverse specific binding modes for a heterogeneous ECM biomaterial.

### 2.3 iECM Components Readily Localize to Sites of Permeable Vasculature and Reduce Vascular Permeability In Vivo

Following material characterization of the iECM components, we investigated their biodistribution and retention in a rat model of MI to determine which component may drive localization and retention in inflamed vasculature in vivo. Following MI, a simulated intracoronary injection was performed with fluorescently tagged complete iECM, high MW, and low MW components at the relative mass‐based concentrations (4 mg/mL complete iECM, 0.76 mg/mL high MW components, 3.24 mg/mL low MW components). To match the relative fluorescence of each of the iECM components, iECM was fluorescently labeled prior to separation by ethanol. Hearts were excised and fluorescently imaged at 1 h and 3 days post‐MI. When images of all scanned tissues are adjusted to the same dynamic ranges, the localization of complete iECM and low MW were more visible compared with saline and high MW (Figure S3a). However, when the high MW and saline were adjusted alone, localization of the high MW was apparent (Figure S3b). This was because of differences in fluorophore conjugation efficiency due to significant differences in free amine content between the components. While the high MW component was approximately 19% by mass of iECM, it only contained 2.6% of the fluorescent dye (Figure S3c). Similarly, the low MW was 81% by mass of iECM and 97.4% of the fluorescent dye (Figure S3c). These values were calculated using the fluorescence of complete iECM after ethanol treatment and no separation. Additionally, the fluorescence values of complete iECM without ethanol treatment was the same as ethanol treatment and no separation. Nonetheless, when evaluating total signal, complete iECM signal was greater than both the high MW and low MW components combined at 1 h (Figure 4a), suggesting improved retention when delivered together. At day 3, the complete iECM still had more signal than both components, further supporting improved retention when the components are delivered together (Figure 4b). After normalization to account for the differences in fluorophore conjugation efficiency, we found that the high MW components localized to the infarct significantly more than the complete iECM or the low MW components at 3 days, though no significant difference were observed at 1 h post reperfusion (Figure 4c,d). These data are in alignment with the QCM data that suggested greater efficiency to our selected substrates by the high MW components alone compared to the low MW components. Additionally, seeing the significantly higher high MW normalized signal at 3 days suggested that the high MW component takes longer to degrade and clear from the infarct, potentially due to its macromolecular structure. These data suggest that the high MW exhibits the greatest binding efficiency and retention in vivo, informing future iECM formulations and bioinspired infusible biomaterial design.

**FIGURE 4:**
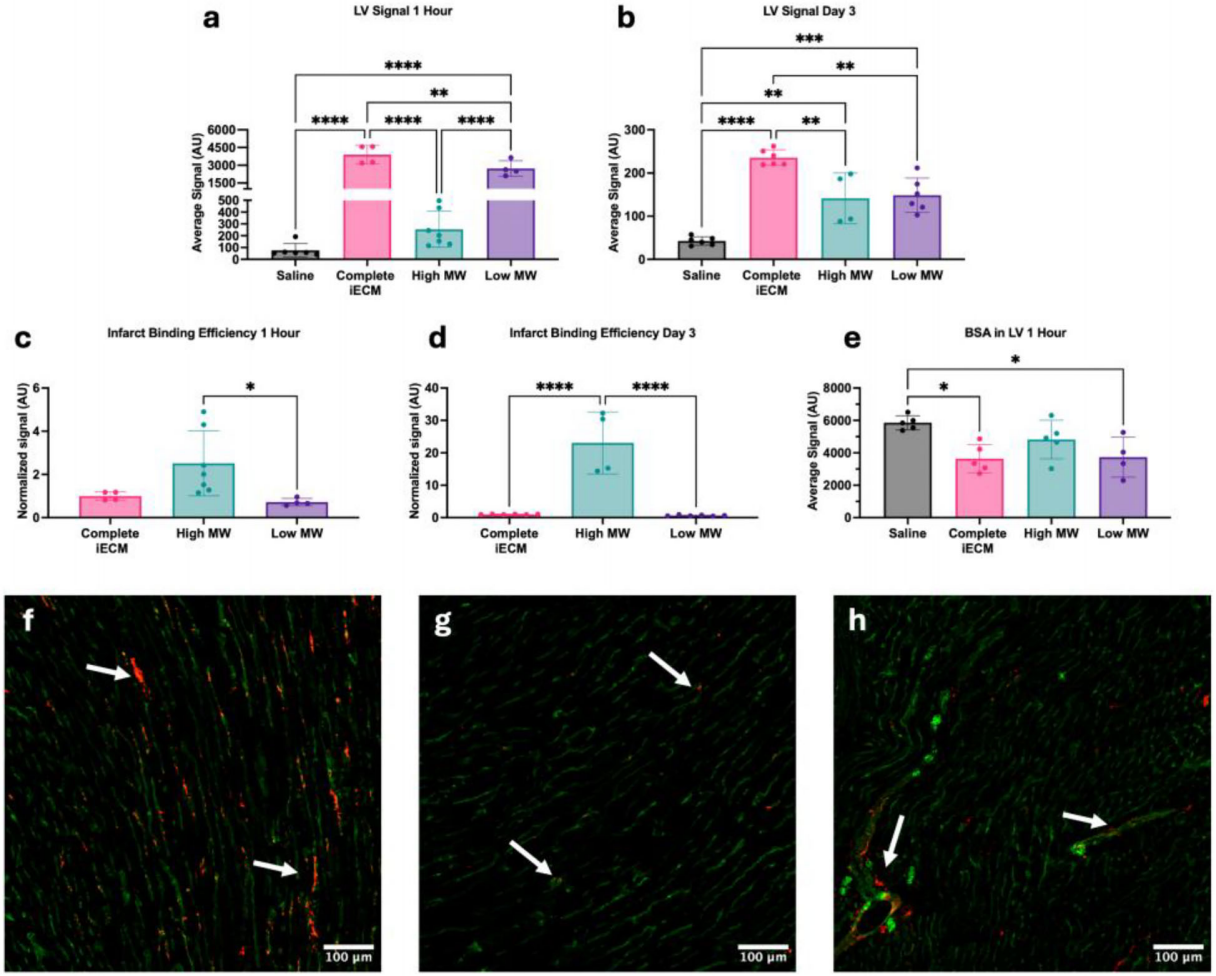
Biodistribution, retention, and effect of the iECM and its fractions on vascular permeability 1 h and 3 days post‐MI. Quantified fluorescent signal of iECM and iECM fractions a) 1 h post‐reperfusion and b) 3 days post reperfusion. The LV retention signal normalized to the fluorophore conjugation efficiency for complete iECM and its fractions at c) 1 h and d) 3 days. e) Vascular permeability of the infarct as determined by residual BSA in LV at 1 h. Immunofluorescent stains of the f) iECM, g) high MW, and h) low MW in red and isolectin for vessels in green within the infarct. White arrows denote example points of colocalization with vasculature. Statistical significance was determined by one‐way ANOVA with Tukey's post‐hoc test (*n* = 4–7 biological replicates). Error bars represent standard deviation. ^*^ *p* < 0.05, ^**^ *p* < 0.01, ^***^ *p* < 0.001, ^****^ *p* < 0.0001.

Post‐MI, inflammation from prolonged ischemia and reperfusion induces endothelial dysfunction, resulting in increased vascular permeability, edema, and additional tissue damage [47, 48]. Previous work demonstrated that iECM reduced vascular permeability following infusion, which is a hypothesized contributor to iECM's therapeutic effects [19]. Using a fluorescently tagged bovine serum albumin (BSA) tracer injected 30 min post‐reperfusion, both complete iECM and the low MW components alone were able to significantly reduce vascular permeability compared to the saline control (Figure 4e). Despite high binding efficiency, high MW components were not significantly different compared to any group (Figure 4e). This suggests that the low MW components are the predominant driver for reducing vascular permeability at the concentrations delivered in iECM.

Via fluorescent staining, we saw colocalization of iECM, high MW, and low MW with endothelial cells within the infarct (Figure 4f–h), confirming their localization to the infarcted vasculature similar to previous results [19]. Overall, these data showed that the iECM components bind to the permeable infarct vasculature. While the low MW components alone were able to reduce vascular permeability soon after reperfusion, the high MW had significantly higher binding efficiency within the infarct at both 1 h and 3 days post‐MI. Altogether, the complete iECM exhibited the highest total signal at both 1 h and 3 day, suggesting improved binding and retention over the individual components.

### 2.4 Delivery of iECM and its Components Result in Transcriptomic Changes Following AMI

Previous work with iECM has demonstrated its ability to induce transcriptomic changes within the infarct [29]. Given that both components appeared to localize to the infarct, we then sought to better understand the role of each of iECM's components on its bioactivity. To this end, we performed single‐nucleus RNA sequencing (snRNAseq) on tissue samples isolated from animals treated with complete iECM, iECM components, or saline 7 days post‐MI since previous work has shown that iECM exhibits the most abundant differences in gene expression at this timepoint [29]. We pooled 4 biological replicate heart tissues into 2 sequencing replicates as was performed previously [29, 49, 50] to help reduce biological variability within each sequencing replicate while maintaining sufficient cell capture to for biological pathway analysis at a reasonable cost. Isolation of iECM and saline groups in this batch of therapeutic treatment was combined with previous data [29] of the same treatment and a different batch to reduce false positive DEG identification of iECM and saline treatment groups and to evaluate the consistency of the same treatment across therapeutic batches. Infarcts from each treatment were quantified to ensure sufficient injury to the myocardium before nuclei isolation; infarct sizes were not significantly different between treatments (Figure S4a–c). The SoupX function was used to remove ambient RNA contamination, and the features and counts were evaluated (Figure S5a,b). The datasets from all treatments were integrated and coarsely clustered. We identified fibroblasts, cardiomyocytes, endothelial cells, macrophages, mural cells (pericytes and vascular smooth muscle cells), proliferating cells, T cells, B cells, endocardial cells, neuronal cells, lymphatic endothelial cells and mesothelial cells (Figure S5c,d) using marker genes previously found in the literature [29]. The relative proportions of each cell type are also shown (Figure S5c) and outlined by replicate in Table S3. After separating the data by cell type, the data was also split by treatment. The saline data was the normalized reference for integration for each treatment. Integration was separate between treatments so that the cells from the other treatments would not impact the integration and bioinformatic removal of contamination and doublets. After contamination and doublet removal, the entire cluster was evaluated for differentially expressed genes (DEGs) between the saline and treatment groups (Tables S4–S12). The upregulated and downregulated DEGs were then analyzed via Gene Ontology (GO) pathway enrichment for biological processes, molecular functions, and cellular components.

#### 2.4.1 iECM and its Components are Robustly Angiogenic

Within the endothelial cell (EC) population (*Flt1^+^/Cdh5^+^)* (Figure 5a–c, Figure S6a–c), iECM upregulation of genes such as *Notch1* [51] and *Plxnd1* (Figure 5a) suggested an angiogenic response, which matches previous findings [29]. When looking at the high MW, significant signs of angiogenesis can be seen (Figure 5b) through upregulation of *Tgfbr1*, *Flt1, Plxnd1, Nrp1*, and *Nrp2* [52]. Additionally, there is integrin upregulation via *Itga1* and *Itga6*, which aids adhesion and migration of circulating cells, chemoattraction via upregulated *Cxcl12*, and differentiation into endothelial progenitors, evidenced with upregulated *Cd34* and *Kdr (Vegfr2)*. The low MW also shows similar signs of angiogenesis via *Flt1*, *Itga6*, *Pecam1*, and *Nrp1* (Figure 5c). Overall, these data suggest a robust angiogenic response across all treatments with signs of endothelial progenitor cell recruitment.

**FIGURE 5:**
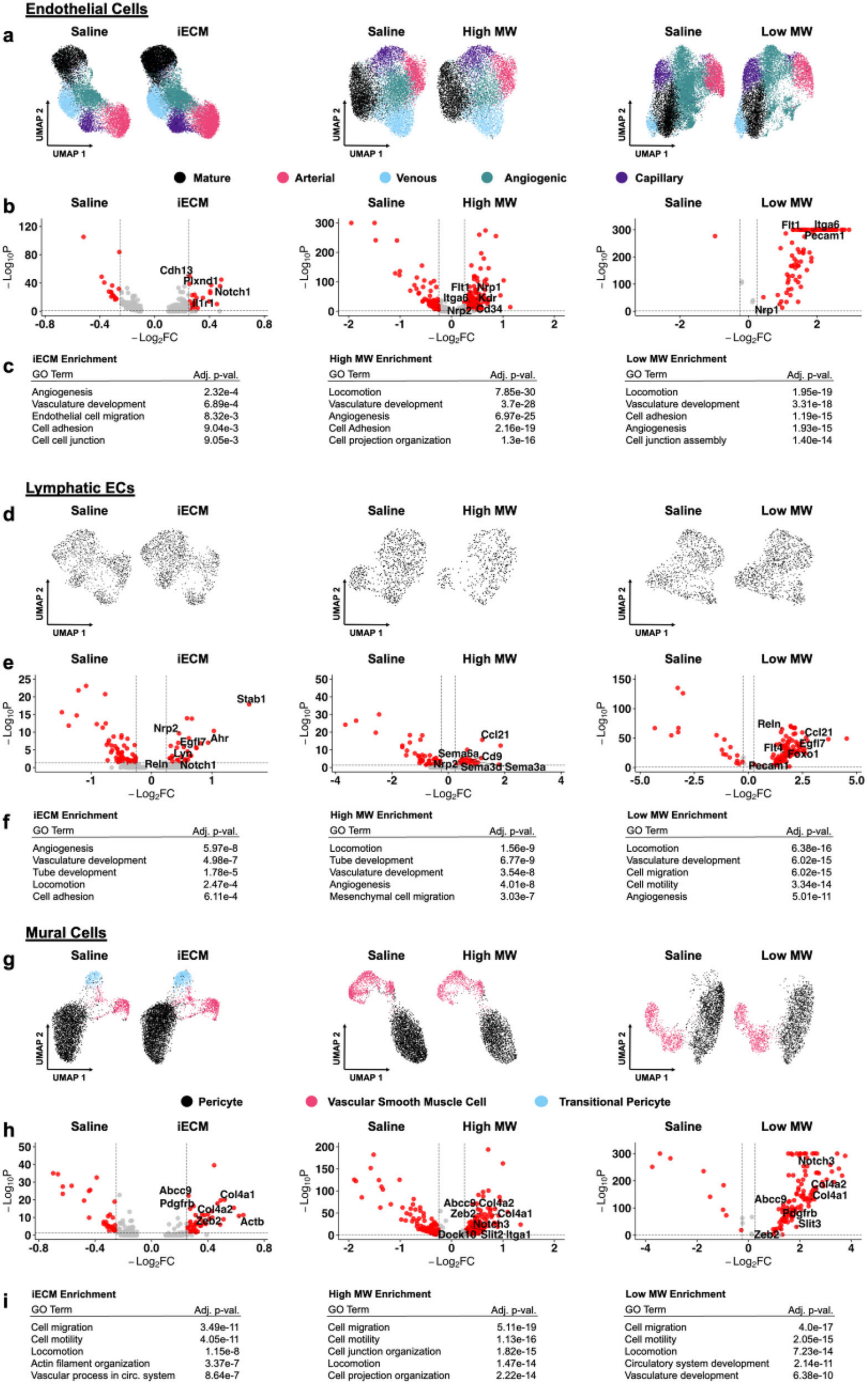
Single nucleus RNA sequencing integration, differential gene expression, and gene ontology enrichment analysis of endothelial cells, lymphatic endothelial cells, and mural cells for iECM, high MW, and low MW compared to saline treatment. The UMAPs, volcano plot, and enriched GO terms for iECM, high MW, and low MW vs. saline for a–c) endothelial cells, d–f) lymphatic endothelial cells, and g–i) mural cells suggest that all treatments resulted in angiogenic and vasculature development‐related responses. Sample size *n* = 8 animals pooled into *n* = 4 sequencing replicates for iECM, *n* = 6 animals pooled into *n* = 3 sequencing replicates for saline, and *n* = 4 animals pooled into *n* = 2 sequencing replicates for high MW and low MW.

Within the lymphatic EC population (*Prox1^+^/Flt4^+^I*) (Figure 5d–f), iECM treatment showed upregulation of *Notch1* and *Egfl7*, which are angiogenic markers [53] matching previous work [29]. Moreover, iECM treated lymphatic ECs also exhibit *Reln*, a lymphoangiocrine that is tied to cardiac growth and repair [54], and *Ahr* which has shown to improve cardiac function [55]. Within the high MW, there was via SEMA signaling and upregulation of *Ccl21*, *Nrp2*, and Cd9 upregulation (Figure 5e), which showed a similar angiogenic response [56, 57, 58, 59, 60]. Within the low MW (Figure 5f), angiogenesis was also seen via *Egfl7* in addition to the lymphoangiocrine *Reln*. There was also robust SEMA signaling and immune cell recruitment via *Ccl21*, *Flt4*, and *Pecam1* and upregulation of *Foxo1* showing signs of mesenchymal cell recruitment [61, 62]. Overall, the lymphatic EC response of all treatments matched the angiogenic response of the endothelial cells in addition to immunomodulation, and lymphoangiocrine signaling.

Within the mural cell subset, which includes pericytes and vascular smooth muscle cells (*Abcc9^+^* or *Myh11^+^*) (Figure 5g–i, Figure S7a–c), all treatments generated upregulation of *Col4a1* and *Col4a2* (Figure 5g–i), which are components of the basement membrane of vasculature [63, 64]. This suggested that mural cells were supporting the stabilization of the vasculature within the infarct due to treatment with iECM and its components. All treatments also showed upregulation of *Zeb2*, which has been linked with cardiomyocyte growth and promotion of angiogenesis [65], and upregulation of *Abcc9* which has been linked with smooth muscle cell development [66]. The high and low MW both showed upregulation of *Notch3* further supporting smooth muscle cell development [67] and *Dock10*, which has been linked with regulation of cardiac function under stress [68]. The low MW also had *Slit3* upregulation, which has been tied to vasculature development [69]. Conversely, all treatments also showed upregulation of *Il34*, which has been linked with macrophage recruitment and increased inflammation post‐MI [70]. The high and low MW also showed *Rock1* upregulation, which revealed signs of reduced endothelial cell development [71]. Overall, these findings suggest that the mural cells from all treatments show clear signs of vasculature support and maturation.

When the vascular cell populations are considered as a whole, iECM and its components exhibit strong signs of angiogenesis and vessel development. Additionally, a variety of subpopulations were identified and conserved across each treatment (Figures S6–S9), which suggests that the therapeutic effects of the individual iECM components was not significantly different.

#### 2.4.2 iECM and its Components Show Signs of Immunomodulation

The major immune cell types captured included B cells (*Cd74^+^/Bank1^+^)*, T cells (*Themis^+^/Bcl11b^+^)* and macrophages (*Mrc1^+^/Csf1r* ^+^ or *Maf^+^/Abca1^+^)* (Figure 6, Figures S8–S10). While neutrophils have been widely implicated in ischemia reperfusion injury [3] and have been a target of interest for immunomodulation post‐MI, they were not studied here since single nucleus RNAseq does not capture neutrophils, but is required for cardiomyocytes. Furthermore, neutrophils peak at 24 h post‐MI and are reduced by day 7 [3].

**FIGURE 6:**
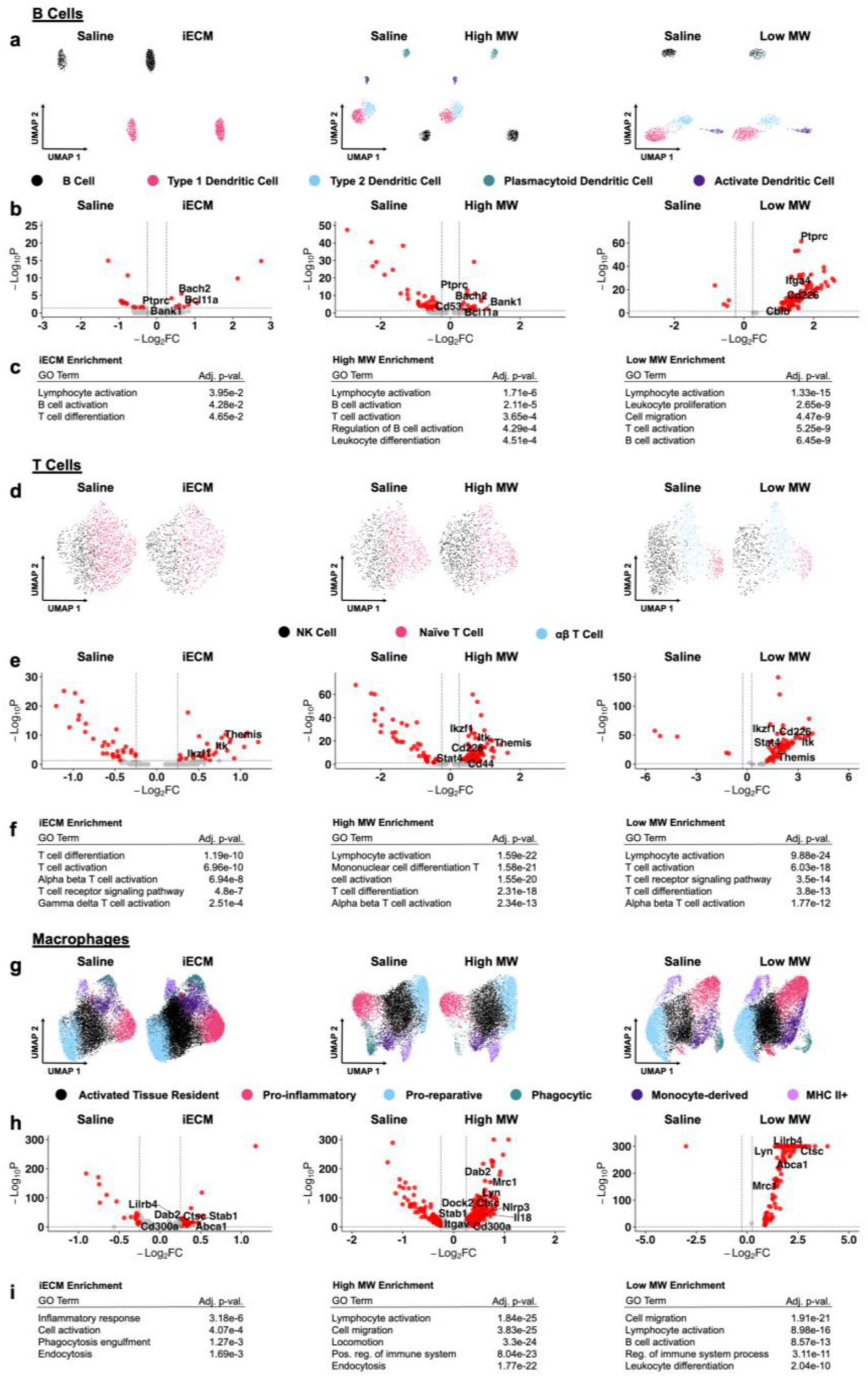
Single nucleus RNA sequencing integration, differential gene expression and gene ontology enrichment analysis of B cells, T cells, and macrophages for iECM, high MW and low MW treatment compared to saline treatment. The UMAPs, volcano plots and enriched GO terms for iECM, high MW and low MW vs. saline for B cells (a–c), T cells (d–f) and macrophages (g–i) respectively. All treatments generated similar responses versus saline via activation of the immune cell type of captured. Overall, the M2‐macrophage response was sustained at day 7 by all treatments vs. saline. Sample size *n* = 8 animals pooled into *n* = 4 sequencing replicates for iECM, *n* = 6 animals pooled into *n* = 3 sequencing replicates for saline and *n* = 4 animals pooled into *n* = 2 sequencing replicates for high MW and low MW.

The B cell population contained other antigen presenting cells like dendritic cells (Figure 6a–c, Figure S8). There were significant B cell activation and development in all treatments via *Bcl11a* and *Ptprc* upregulation. In addition, there was *Bank1* expression for iECM and high MW groups and *Cd53* in the high MW (Figure S6b) further supporting activation [72]. The B cells of iECM and high MW also showed upregulation of *Bach2*, which has been linked to reduced cardiac hypertrophy [73]. The low MW also showed signs of B cell development via *Bcl11a*, *Ptprc*, *Itga4* and *Cd226*. There was also *Cblb* upregulation suggesting a potential reduction in inflammatory cascades [74].

The T cell population, which also contained an NK cell subpopulation, in all treatments suggested similar activation and differentiation via upregulation of *Themis*, *Bcl11b*, and *Ikzf1* (Figure 6d–f, Figure S9). Additionally, the high MW exhibited *Cd44* upregulation, which has been linked with infarct healing via immunomodulation [75] as well as *Stat4*, which is linked to alleviation of injury post‐MI [76]. Despite this, a pro inflammatory T cell phenotype existed seen through *Itk* expression [77] across all treatments and *Cd226* expression [78] in the high and low MW. Overall, this data suggested that while iECM and its components do not fully ablate the inflammatory response post‐MI, all treatments generated signs of an immunomodulation toward a pro‐repair response.

Within the macrophage subset (Figure 6g–i, Figure S10a–c), there was a mixed expression of proinflammatory M1‐like and pro‐reparative M2‐like macrophages. Additionally, similar to previous work, monocytes were not capture at this timepoint suggesting the polarization of these cell types by day 7 [29]. For iECM treatment (Figure 6g), pro‐reparative genes [79, 80] are expressed like *Stab1* [79] and *Dab2*, alongside pro‐inflammatory genes [81, 82, 83, 84, 85] like *Lilrb4*, *Abca1*, *Cd300a* and *Ctsc*. In the high MW treatment (Figure 6h), upregulation of *Lilrb4, Nlrp3*, *Tlr7*, *Il18*, *Dock2*, *Ctsc*, *Cd300a*, and *Abca1* signified an M1 macrophage presence [81, 82, 83, 84, 85] while upregulation of *Mrc1*, *Dab2*, *Stab1*, *Itgav, Lyn*, and *Csf1r* supported a significant M2‐macrophage population [29, 86]. The low MW treatment (Figure 6i) showed M1 gene expression via *Ctsc*, *Lilrb4* and *Abca1* as well as M2 gene expression via *Lyn* and *Mrc1* (Figure 6i). This mixed M1/M2 macrophage response has been previously demonstrated, both with our earlier work with iECM [29] and with other decellularized ECM materials [87, 88]. Altogether, these results reveal a robust immunomodulatory response across multiple immune cell types as a result of iECM treatment and its components, with much of the transcriptional profiles matching previous reports [29].

#### 2.4.3 iECM and its Components Promote a Pro‐Regenerative Phenotype

Within the cardiomyocyte subset (*Ryr2^+^/Tnni3k^+^*) (Figure 7a–c, Figure S11a–c), the overall phenotype across treatments suggests a shift toward cardioprotection, although some fibrotic and hypertrophic response still exists. With iECM treatment (Figure 7a), upregulation of *Vegfa Ddah1*, *Srsf3, Ndrg4*, and *Ddx17*, suggest cardiac angiogenesis and preservation of systolic function [89, 90, 91, 92]. Conversely, *Rnf207* was also upregulated with iECM treatment, which has been tied to cardiac hypertrophy [93]. With high MW treatment (Figure 7b), there was upregulation of *Erbb4*, which has been linked with mesenchymal cell myocardial repair [94]. In addition, the high MW had upregulation of *Corin, Tbx20*, *Pde3a*, and *Nrp1*, which have been linked with reduced cardiomyocyte apoptosis [95, 96, 97, 98, 99] and upregulation of *Fgf12* and *Fgf13*, which has been linked with reduced cardiac remodeling and reduced inflammation [100, 101]. With low MW treatment (Figure 7c), similar to the high MW, there was also upregulation of *Erbb4, Corin*, *Tbx20*, *Fgf12, Fgf13*, and *Pde3a*. Additionally, the low MW treatment had upregulation of *Fgf1*, which has been linked with cardiac regeneration [102].

**FIGURE 7:**
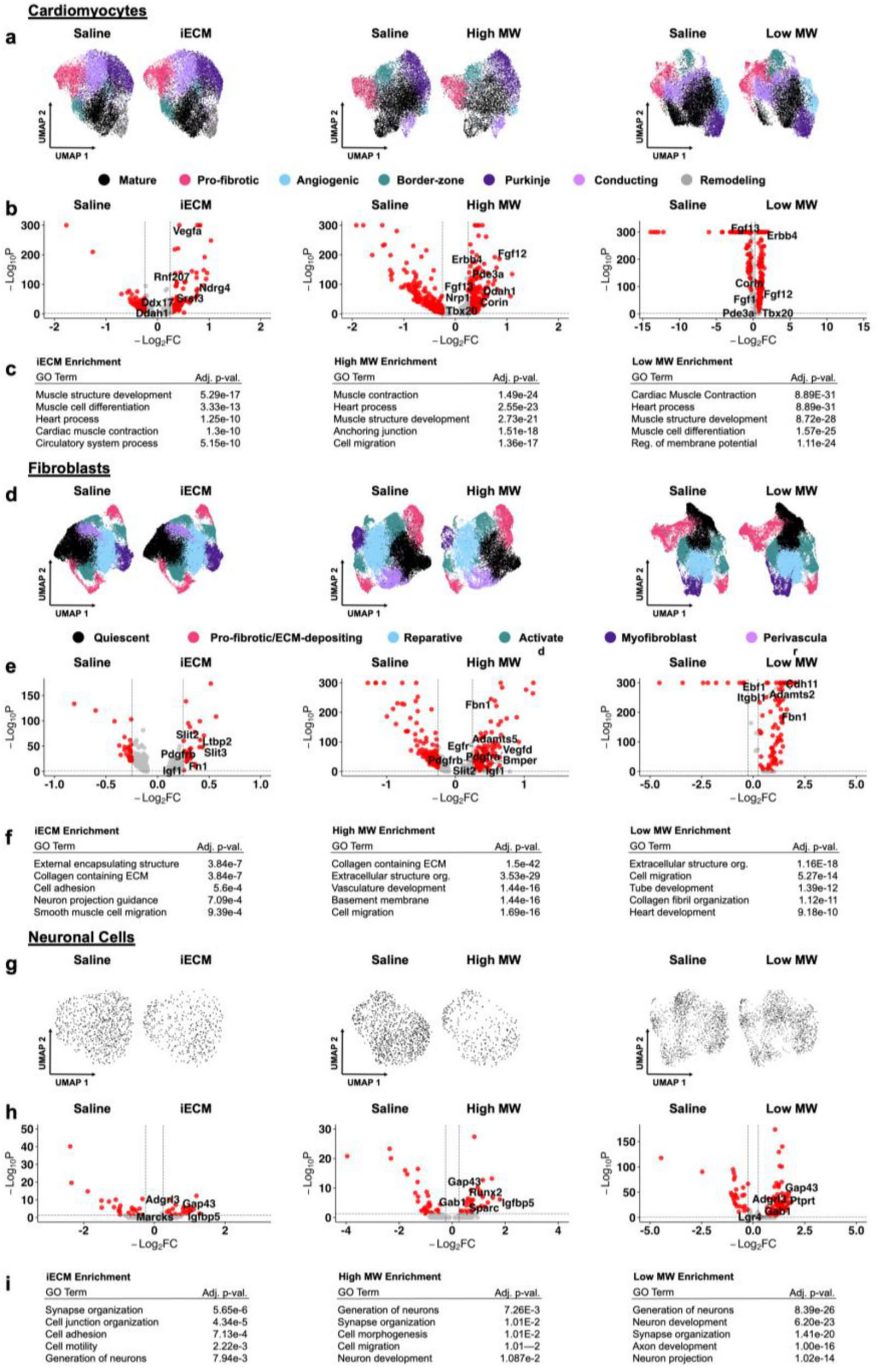
Single‐nucleus RNA sequencing integration, differential gene expression, and gene ontology enrichment analysis of cardiomyocytes for iECM, high MW, and low MW compared to saline treatment. The UMAPs, volcano plots, and enriched GO terms for iECM, high MW, and low MW vs. saline for a–c) cardiomyocytes, d–f) fibroblasts and g–i) neuronal cells. The overall response of the cardiac function‐related cell types was cardioprotection. Sample size *n* = 8 animals pooled into *n* = 4 sequencing replicates for iECM, *n* = 6 animals pooled into *n* = 3 sequencing replicates for saline, and *n* = 4 animals pooled into *n* = 2 sequencing replicates for high MW and low MW.

Within the fibroblast subset (*Postn^+^/Dcn^+^*) (Figure 7d–f, Figure S12a–c), DEGs across treatments consisted of genes implicated within fibroblast activation and cardiac fibrosis as well as with cardioprotection (Figure 7a–c). Within the iECM treatment group (Figure 7a), *Slit3*, *Ltbp2*, and *Fn1* were upregulated, which has been linked with cardiac fibroblast activation and fibrosis [103, 104]. There was also upregulation of *Slit2*, *Igf1* and *Pdgfrb*, which has been linked with reduced fibrosis [105], cardiomyocyte proliferation [106, 107, 108] and reduced inflammation [105, 109], cardiomyocyte proliferation [106, 107, 108], and reduced inflammation [109]. Within the high MW (Figure 7b), there was similar fibroblast activation [110, 111, 112, 113, 114, 115, 116] with upregulation of *Egfr*, *Itgbl1*, *Fbn1, Vegfd, Adamts5*, and *Bmper* while also showing signs of cardioprotection [105, 106, 107, 108, 109, 117] via upregulation of *Pdgfra, Pdgfrb, Igf1*, and *Slit2*. Within the low MW (Figure 7c), upregulation of *Cdh11, Fn1*, *Itgbl1* and *Adamts2* pointed toward fibroblast activation [118] while *Ebf1* upregulation pointed toward reduced fibrosis [119]. The response within the fibroblast subsets for all treatments showed signs of fibroblast activation and cardioprotection. This shows that while iECM, high MW, and low MW treatment are able to induce evidence for an increased cardioprotective phenotype, the canonical fibroblast activation and fibrotic pathways are not ablated.

Within the neuronal cell subset (*Cadm2^+^/Ncam2^+^*) (Figure 7g–i), all treatments showed signs of neuron development and proliferation via *Gap43* upregulation [120, 121]. With iECM (Figure 7g), upregulated genes included *Marcks*, which is linked with nerve regeneration [122], *Igfbp5* upregulation, which is tied to cardiomyocyte survival [123], and *Adgrl3*, which has been linked with tissue development [124]. With high MW (Figure 7h), *Igfbp1* upregulation was also seen alongside *Sparc* and *Gab1*, which has been linked with fibrosis and cardiomyocyte apoptosis [125]. Within the low MW (Figure 7i), *Gab1* and *Adgrl3* upregulation were seen alongside *Ptprt* [126] and *Lgr4* [121], which has been linked to neuronal development. Overall, the expected neuronal sprouting genes typical post‐MI is seen via *Gap43* expression. In addition, evidence suggests neuronal cell development within all treatments.

#### 2.4.4 iECM and its Components Exhibit Similar Gene Ontology Enrichment

iECM, high MW, and low MW exhibited a variety of gene expression that all pointed toward a similar phenotype across all major cell types. When these DEGs were analyzed for gene ontology (GO) pathway enrichment, many conserved pathways were found across the 3 treatments with subtle differences (Figure 8). All treatments enriched for angiogenesis in both endothelial and lymphatic endothelial cells. Within mural cells, all treatments enriched for cell migration, suggesting that all the treatments resulted in strong vasculature development signatures. Within immune populations, all treatments enriched for lymphocyte activation within B and T cells as well as macrophage activation and endocytosis. The fibroblast response was equally similar, showing signs of activation through cell adhesion and ECM‐related pathways. Finally, the pathway enrichment for cardiomyocytes and neuronal cells were consistently pro‐repair for all treatments, suggesting cardioprotection and the development of neurons. Many of these observations of the angiogenic, immunomodulatory and cardioprotective effects of iECM are corroborated by previous transcriptomic and histological evidence [19, 29].

**FIGURE 8:**
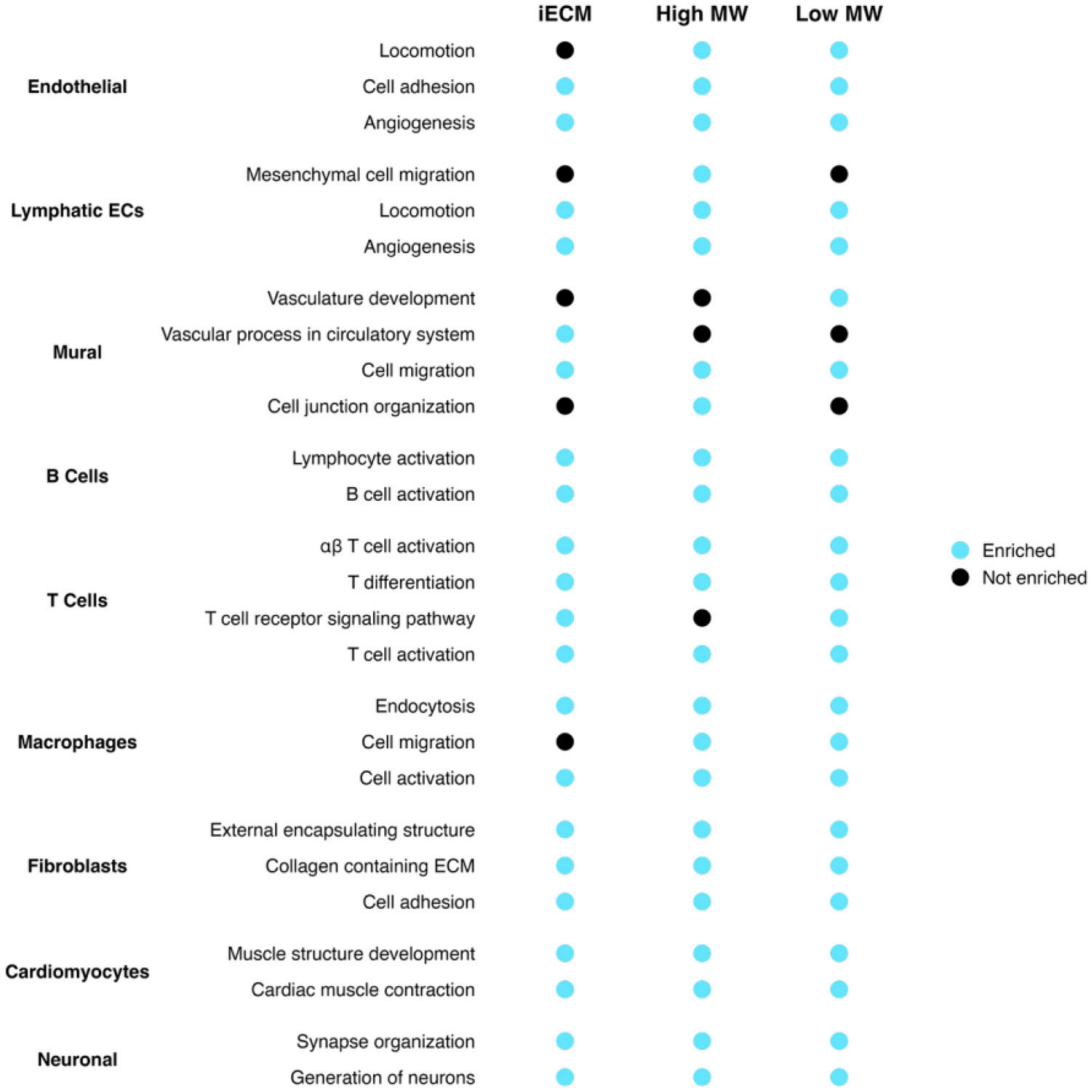
Summary of GO enrichment for each treatment vs. saline per cell type. The enriched pathways were similar between the treatments, suggesting an angiogenic response for vascular‐related cell types, immune cell activation, and pro‐reparative phenotypes for cell types related to cardiac function. Sample size *n* = 8 animals pooled into *n* = 4 sequencing replicates for iECM, *n* = 6 animals pooled into *n* = 3 sequencing replicates for saline, and *n* = 4 animals pooled into *n* = 2 sequencing replicates for high MW and low MW.

The GO summary shows that both components of iECM exhibit pro‐reparative effects that are conserved when delivered individually. Additionally, the DEGs were not confined to a single component, indicating that the bioactivity of the high‑ and low‑MW components overlap. When contextualized with the compositional overlap seen in the mass spectrometry results (Figure 2c,d), these data suggest that the differences in morphology and biochemical nature may drive the differences in binding efficiency in vitro and in vivo while the composition drives the downstream gene expression. However, since the two components do have varying abundances of a similar composition, there still exists subtle differences in the differential gene expression and pathway enrichment analysis, particularly illustrated in the vascular cell type response generated by the high MW. Overall, these gene expression data show that the composition of ECM biomaterials drive the reparative response regardless of their morphology and biochemical features highlighting the intrinsic pro‐reparative nature of ECM biomaterials.

## 3 Conclusion

We were able to extensively characterize the material properties and biological response for iECM and its components through methods not previously utilized for ECM biomaterials. Within iECM, we identified high and low MW components that are biochemically and morphologically distinct with differences in in vitro binding to ECM components and cellular receptors. Proteomic analysis revealed both components were comprised of peptides from similar proteins, though the relative abundance differed between the two. Evidence from QCM measurements also demonstrated that the high and low MW components interact with one another, further suggesting binding interactions with exposed ECM. These differences seen in vitro translate to differences in behavior after administration following MI. While the low MW components alone were able to significantly reduce vascular permeability at the 1 h timepoint, the high MW components bound to the compromised endothelium more efficiently. However, the combination of both components resulted in increased total signal, suggesting the importance of the total composition of iECM on acute localization. snRNA‐seq showed that across all major cell types, the angiogenic, immunomodulatory, and pro‐regenerative phenotypes were similar across iECM, high MW, and low MW treatment versus saline. Both the DEGs and conservation of GO enrichment between therapeutic treatment groups further highlighted the interrelated behavior of the two iECM components. Collectively, these data suggest that the localization of both iECM components allows them to engage vascular‐related cell populations, thereby shaping the immune response and impacting cardiomyocytes, key determinants of myocardial function.

While these findings complement current sentiment that ECM biomaterials are therapeutic through their heterogeneity, deep analysis of iECM components and their characteristics also reveals new approaches to designing infusible biomaterials. Specifically, this work demonstrates that the flexible nanofibrillar shape seen in the TEM, combined with multivalent, multitargeted binding as shown via QCM confers high specificity for and retention in vasculature at sites of inflammation. These findings highlight material attributes that can be leveraged for designing and improving targeted intravascular biomaterials either via synthetic biomimicry or processing modifications to biologically derived materials.

## 4 Experimental Section

### 4.1 Manufacturing of iECM

Porcine‐derived LV myocardium was decellularized according to previously published protocols [19]. The LV myocardium from whole porcine hearts (Collagen Solutions) was isolated, with the endocardium and epicardium removed. Isolated LV myocardium was ground, passed through a ¼ inch stainless steel grate, and decellularized with 1% sodium dodecyl sulfate (SDS, Fisher Scientific) in PBS for 4 days, followed by 24 h of water rinsing. Material was then lyophilized, milled, and passed through a 60‐mesh screen.

Milled decellularized ECM was digested at 10 mg/mL in 1 mg/mL pepsin (Millipore Sigma) in 0.1 m HCl for 48 h. Following digestion, the solution was neutralized with 1 m NaOH, diluted to 6 mg/mL in 1X PBS, and centrifuged at 14 000 RCF at 4°C for 45 min. The supernatant was collected, dialyzed against pure water for 48 h, and lyophilized. Dry material was resuspended at approximately 16 mg/mL in PBS, filtered through a 0.22 µm filter, aliquoted, lyophilized and stored at −80°C until use. Prior to use, iECM was resuspended at 4 mg/mL in sterile water and stored on ice until use. iECM components were separated out through resuspension in 50% ethanol, causing the high MW to precipitate out. Solutions were centrifuged at 14000 RCF for 10 min to pellet the high MW components. This precipitation and centrifugation method was repeated once. Pellets and supernatants were lyophilized and resuspended with PBS and sterile water, respectively, before use. For experiments comparing complete iECM, high MW components, and low MW components, complete iECM underwent 50% ethanol precipitation but was lyophilized and resuspended prior to use with no centrifugation or speration.

### 4.2 Characterization of iECM Molecular Weight and Relative Composition Analysis

For quantitative molecular weight analysis, iECM was analyzed with size exclusion chromatography (SEC) using a Bio‐Rad NGC Quest 10 Plus fast protein chromatography system. 200 µL of 4 mg/mL iECM was flowed at 1 mL/min through a Bio‐Rad Enrich SEC 650 column (10 × 300 mm) with a separation range of 5–650 kDa. Mobile phase molecules were detected by absorbance at 215 nm. Relative composition of iECM was determined by integrating the area under the curve and determining the relative area of each of the peaks. Apparent molecular weight was determined by constructing a molecular weight curve using a protein gel filtration standard (Bio‐Rad) with a range of 650–5 kDa. Confirmation of iECM fractionation was done using SEC and through SDS‐PAGE. A 4%–12% Bis‐Tris gel (Invitrogen) and a broad‐range pre‐stained protein ladder (Abcam) were used for PAGE. Protein bands were visualized using an Imperial protein stain (ThermoFisher).

### 4.3 Characterization of iECM Components

#### 4.3.1 Biochemical Characterization

Free amines in iECM components were determined using an o‐Phthaldialdehyde reagent assay (ThermoFisher) and compared against glycine standards. Free thiols were determined using a thiol detection assay kit (Cayman Chemical) and compared against cysteine standards. Sulfated glycosaminoglycans were determined using a 1,9‐dimethylmethylene blue assay and compared against chondroitin sulfate standards [127]. dsDNA was isolated using a NucleoSpin kit (Macherey‐Nagel) and quantified using a PicoGreen Fluorescent reporter (Invitrogen). To determine fluorophore conjugation efficiency to iECM components, 4 mg/mL complete iECM was labeled with 10 mg/mL Vivotag‐S 750 (VT‐750, Perkin Elmer) at a volume ratio of 1:100. Labeling was done at room temperature for 1 h, after which iECM was separated into components as described above and lyophilized. Dried labeled iECM and iECM components were resuspended at their original volumes and their fluorescence was determined with a UV–vis spectrophotometer plate reader. Fluorophore labeling efficiency was determined by comparing fluorescence of components to the fluorescence of complete iECM.

#### 4.3.2 Mass Spectrometry Proteomics

iECM was subjected to trypsin digestion using S‐Trap micro spin columns (Protifi #C02‐micro) according to the manufacturer's protocol. In brief, 2x S‐Trap lysis buffer was added to each sample to reach 1X concentration. Samples were reduced using Tris (2‐carboxyethly) phosphine (TCEP), alkylated with 2‐chloroacetimide (ClAA), and digested with trypsin (1:100 enzyme:substrate ratio) for approximately 15 h at 37°C. Digested peptides were desalted using Pierce C18 spin columns (Thermo Fisher #84850) according to the manufacturer's protocol.

Digested peptides (200 ng) were loaded onto individual Evotips and separated on an Evosep One chromatography system (Evosep, Odense, Denmark) using a Pepsep column, (150 µm inter diameter, 15 cm) packed with ReproSil C18 1.9 µm, 120Å resin. With a “30 samples per day” LC gradient, the sample was analyzed on a timsTOF Pro mass spectrometer (Bruker Daltonics, Bremen, Germany) via the nano‐electrospray ion source (Captive Spray, Bruker Daltonics) in PASEF mode. The ramp time was 100 ms and 10 PASEF MS/MS scans per topN acquisition cycle were acquired. MS and MS/MS spectra were recorded from m/z 100 to 1700 and ion mobility scanned from 0.7 to 1.50 Vs/cm^2^. Precursors for data‐dependent acquisition were isolated within ± 1 Th and fragmented with an ion mobility‐dependent collision energy, which was linearly increased from 20 to 59 eV in positive mode. Low‐abundance precursor ions with an intensity above 500 counts and below 20 000 counts were repeatedly scheduled and otherwise dynamically excluded for 0.4 min.

Data was searched via MSFragger v4.0 via FragPipe v21.1 against the UniProt protein database restricted to *Sus scrofa* with added common contaminant sequences [128] (46 285 total sequences, downloaded 1/8/25) [129]. Precursor tolerance was ±15 ppm and fragment tolerance was ±25 ppm. Enzyme cleavage was semi‐specific trypsin. Fixed modifications were carbamidomethyl (C). Variable modifications were oxidation (M), oxidation (P) (hydroxyproline), deamidation (NQ), Gln→pyro‐Glu (N‐term Q), and acetyl (protein N‐terminus). IonQuant v1.10.12 was used for label‐free quantification with match‐between‐runs enabled and default parameters. Results were filtered to 1% FDR at the peptide and protein level.

After data was searched, positively filtered results were matched to 3 core matrisomes and sorted. The signal intensities were summed by matrisome categories and plotted to show the relative signal of each category within high MW vs low MW. Protein and peptide overlap were calculated using the list of positively identified proteins for each replicate of high MW and low MW, irrespective of signal intensity proportion. First, for each replicate, the number of proteins identified with a non‐zero signal was summed. From the list of non‐zero proteins for that replicate, any protein within that list that was not found in any of the other 3 replicates of the other component were considered unique to that component. E.g., proteins of high MW replicate 1, were compared to all 3 low MW replicates. This was performed for all replicates of high MW relative to all 3 low MW replicates and vice versa. The overlap percentages were calculated for each replicate and averaged. This was also performed for the peptide overlap analysis bewteen high MW and low MW results.

#### 4.3.3 Transmission Electron Microscopy

First, glow discharged, 200 mesh, Formvar carbon‐copper TEM grids were inverted onto droplets of iECM, high MW, and low MWs for 2 min. Grids were washed 3 times for 30 s using 0.22 µm‐filtered double‐distilled water and stained with 2% uranyl acetate for 1 min, and finished with a 5 s blot. Samples were air‐dried before imaging on a Tecnai G2 Spirit BioTwin (FEI) operated at 80 KeV and equipped with a bottom‐mounted 4kx4k Eagle camera. Images were collected at 2.9, 6.8, 11, and 30kx.

#### 4.3.4 Quartz Crystal Microbalance

Frequency and dissipation data was collected under flow conditions on a QCM‐D QSense Explorer (Biolin Scientific). Gold coated chips were used (Biolin Scientific QSX 301). Prior to experiments, chips were soaked in 2% sodium dodecyl sulfate in water at 70°C for 2 h. Chips were washed 5 times with room‐temperature deionized water, dried, and stored in the original boxes. Prior to experiments, chips were cleaned within a UV/ozone cleaning chamber for 10 min.

N‐hydroxysuccinimide functionalized lipid suspensions (NHS‐DPPC) were produced similar to previous reports [41]. Equal mass (15 mg) of palmitic acid N‐hydroxysuccinimide ester (Sigma P1162) and 1,2‐dipalmitoyl‐sn‐glycero‐3‐phosphocholine (Sigma P4329) were mixed with 15 mL of 0.1 m MES [2‐(2‐morpholino) ethanesulfonicacid] buffer (pH 6.0). Lipid solutions were heated to 60°C and mixed. Lipid suspensions were aliquoted and frozen in −80°C until use.

For binding interaction between high MW and low MW components, the high MW and low MW were coated at a concentration of 5 mg of ECM/mL to ensure consistent exposure of the components during conjugation.

Basal lamina ECM components and endothelial cell surface receptor protein solutions were diluted to 0.1 mg/mL from the stock using 1x phosphate‐buffered saline (1x PBS). Solutions were aliquoted and frozen at −80°C until use. ECM components used were type I collagen (Advanced BioMatrix 5005100 mL 3 mg/mL), type IV collagen (Cultrex Mouse Collagen IV 0.5 mg/vial 3410‐010‐01), laminin 111 (Gibco 1.2 mg/mL 23017015), and laminin 521 (LA5‐H5215‐500ug). Cell surface receptor proteins used for conjugation were P‐selectin (Acro 50‐165‐8924 – 100 ug), integrin α1β1 (Acro 50‐253‐4563 – 100 ug) and ICAM/CD54 (MedChemExpress HY‐P7262).

Antibodies used for validation of ECM and cell surface receptor protein conjugation were diluted to 50 ug/mL using 1x PBS. ECM antibodies were type I collagen (Invitrogen PA5‐29569), type IV collagen (Invitrogen PA1‐85320), laminin 111 (Biosensis R‐1808‐100), and laminin α5 antibody (Abnova H00003911‐D01P). Cell receptor antibodies were P‐selectin (Invitrogen 12‐0626‐82), integrin α1 (Invitrogen MA5‐55280), and ICAM/ (Invitrogen 500‐P287‐100UG).

Flow experiments were carried out at 100 uL/min unless otherwise noted. A 22°C baseline was collected with DI water. NHS‐DPPC was introduced for 5 min followed by a 5 min 1x PBS wash. ECM or cell surface receptor proteins were introduced for 10–20 min at 50 uL/min for conjugation, followed by a 1x PBS wash at 100 uL/min.

For antibody assay validation, the temperature was maintained at 22°C. After the 1x PBS wash following conjugation, a 1 mg/mL bovine serum albumin (BSA) (Geminin Bio 50‐753‐3052) solution in 1x PBS was used for blocking, followed by a 5 min 1x PBS wash. The antibody was introduced at 25 uL/mL followed by a final 1x PBS wash.

For the binding assays of high MW to low MW and vice versa, following the 1x PBS wash after conjugation, the temperature was changed to 37°C. After equilibrium, BSA was used for blocking, followed by a 5 min 1x PBS wash at 100 uL/min before the high MW or low MW (∼1.9 mg of ECM/mL and ∼8.1 mg of ECM/mL respectively) was flowed at 100 uL/min for 10 min followed by a final 1x PBS wash.

For iECM, high MW and low MW component binding experiments, following the 1x PBS wash after target conjugation, the temperature was changed to 37°C. At 37°C equilibrium, BSA was used to block, followed by a 5 min 1x PBS wash at 100 uL/min. Then, the iECM, high MW components or low MW components (10 mg of ECM/mL, ∼0.76 mg of ECM/mL and ∼3.24 mg of ECM/mL) or non‐specific peptide controls (∼8.1 mg/mL) of neutral charge poly‐L‐proline <10 kDa (Sigma P2254‐100MG) and positive charge poly‐L‐lysine <15 kDa (Sigma P6516‐500MG) were flowed at 100 uL/min for 10 min followed by a final 1x PBS wash.

Frequency and dissipation data was exported from the QCM‐D control software as a text file. The data was imported into the NBS‐QCMAnalysis software (Nanobiosensorics Laboratory). Baselines were created using equilibrium timepoints, e.g. the 1x PBS wash after BSA blocking. After baselining, the timepoints of interest were isolated, exported, and the differences in the equilibrium frequencies for the third overtone were extracted and calculated for semiquantitative comparison. All conditions were performed in triplicate.

### 4.4 Testing iECM and iECM Components in Rat Model of MI and I/R Injury

All procedures in this study were approved by the Committee on Animal Research at the University of California San Diego and in accordance with the guidelines of the Association for the Assessment and Accreditation of Laboratory Animal Care (A3033‐01).

#### 4.4.1 Rat Model of MI and I/R Injury

To induce MI, adult female Sprague–Dawley rats (225–250 g) were used as previously described [19, 86]. To induce acute MI, the left coronary artery was accessed via a thoracotomy and ligated for 35 min with a suture. Following ischemia, the suture was released to restore blood flow. For simulated intracoronary infusion, the aorta was clamped and the 200 µL of treatment (saline, 4 mg/mL iECM, 0.76 mg/mL high MW components, 3.24 mg/mL low MW components) was injected into the LV lumen using a 30 G needle. Infusions were performed within 10 min of reperfusion. Animals were randomly assigned treatment groups.

#### 4.4.2 Biodistribution of iECM and iECM Components

To determine biodistribution and LV localization of iECM and iECM components, iECM was labeled with VT‐750 at a ratio of 1:100 by volume. iECM was labeled prior to fractionation with ethanol to preserve the relative labeling ratio found in complete iECM. Labeled material was delivered in the rat model of MI as previously described [19]. At time points of 1 h and 3 days, rats were euthanized via an overdose of sodium pentobarbital via intraperitoneal injection and perfused with PBS to remove residual iECM in the blood. Heart and satellite organs (lungs, kidneys, spleen, liver) were extracted, with the heart sliced into 6 transverse slices. Heart slices and satellite organs were scanned with an Odyssey CLx (LICORbio) near infrared scanner to determine total signal.

#### 4.4.3 Effects on Vascular Permeability

To determine the effects of iECM components on vascular permeability, iECM and iECM components were delivered in a rat model of MI as described above. At time points of 30 min and 3 days post‐reperfusion, 1 mL of 0.06 mg/mL bovine serum albumin (BSA) labeled with Alexa Fluor 680 (ThermoFisher) was administered intravenously through the tail vein. 30 min after delivery, animals were euthanized and perfused to remove residual labeled BSA. Hearts were extracted and sliced into 6 transverse slices. Heart slices were scanned as described above to determine the amount of BSA that extravasated into myocardium using total signal.

#### 4.4.4 Single Nucleus RNA Sequencing

Rats underwent the MI I/R injury model as described in 5.4.1. Treatment groups included saline, complete iECM, high MW and low MW. Animals were harvested 7 days post‐MI. Heart tissue was processed as previously described [86]. In short, hearts were cut into 6 transverse sections. The LV free wall from even sections were isolated, chopped and flash frozen in liquid nitrogen and stored at −80°C before nuclei isolation. Mid infarct sections underwent hematoxylin and eosin staining and brightfield imaging (Olympus VS200). Infarcts were quantified using QuPath by (1) tracing the infarct, (2) the LV free wall with septum and not RV free wall, and (3) the LV lumen. The infarct percentage was then calculated. Hearts below 15% were not used.

Nuclei isolation was performed similar to previous studies [50, 130]. Heart tissue from 4 animals per treatment were pooled into 2 biological replicate samples. Approximately 60 mg of pooled tissue was resuspended in nucleus lysis buffer (Sigma NUC101) with RNase OUT inhibitor (Invitrogen 10777019). Samples were minced on wet ice using scissors (FST 14058‐11) before homogenization with a dounce grinder (Sigma D8938). An additional 1 mL of lysis buffer was added, and samples were incubated for 10 min on ice prior to filtration through 100, 50, and 20‐µm cell strainers (CellTrics NC1037263, NC9491906, NC9699018). Samples were pelleted at 1,000 g for 5 min at 4°C and resuspended in 2 mL of sucrose gradient buffer. Nuclei were pipetted on 4 mL of sucrose gradient buffer before being pelleted at 1000 g for 10 min at 4°. The pellet was washed with nucleus storage buffer, pelleted, washed with 5% BSA in 1x PBS, pelleted, and resuspended in 200 µL of 2% BSA and 5 µL of DAPI. Nuclei were diluted with trypan blue, loaded onto a hemocytometer (Bulldog‐Bio DHC‐N21), and intact nuclei were counted using a brightfield phase‐contract (Keyence BZ‐X).

Nuclei were then subject to the 10x Chromium Universal 3’ Gene Expression v4 protocol. Paired‐end sequencing was performed on an Illumina NovaSeq instrument. Low‐level analysis, including demultiplexing, mapping to a reference transcriptome and eliminating redundant unique molecular identifiers (UMIs), was performed with the Cell Ranger 9.0.1 pipeline for the 10X samples.

#### 4.4.5 Quality Control, Normalization, Integration, Coarse Clustering, Subset Analysis and GO Enrichment Analysis

SN RNA‐seq data was analyzed using methods adapted from previously [50, 130] in addition to the latest version of Seurat 5.2.0 [131, 132, 133, 134, 135]. After alignment, the counts matrices were adjusted using SoupX. Cells with a minimum of 200 uniquely expressed genes and a maximum of 2500, with less than 5% mitochondrial content, were isolated. Mitochondria‐related genes were then removed. The data was normalized using log normalization. Cells were analyzed unintegrated before integrated analysis using IntegrateLayers(), RPCA reduction, and a resolution of 0.1 for the coarse clustering. The coarse clusters were identified for general cell type populations and subset for further analysis.

Subset clusters were isolated in groups of two treatments and re‐normalized using SCTransform, clustered and subject to FindAllMarkers(). Clusters containing non‐endogenous gene markers were contaminants and thus removed. This subset cleaning process was performed iteratively. With cleaned subsets, DEGs were calculated for treatment vs. saline, with adjusted *p* value < 0.05, log fold change threshold of 0.25, min.pct = 0.25 to define the DEGs. These DEGs were plotted on volcano plots with positive and negative DEGs set as inputs for GO analysis via GSEA. The most relevant pathways were selected and organized by FDR/adjusted *p*‐value.

### 4.5 Statistical Analysis

Statistical analysis was performed on GraphPad Prism10. For analysis of two groups, a two‐tailed unpaired *t*‐test with Welch's correction was performed. For analysis of multiple groups, a one‐way ANOVA with post hoc Tukey's test was performed. For analysis of multiple groups at multiple conditions, a two‐way ANOVA with post hoc Tukey's test was performed. Significance was accepted at p<0.05.

## Author Contributions

**Michael Nguyen**: Conceptualization, Methodology, Validation, Formal analysis, Investigation, Data curation, Visualization, Writing – Original draft preparation, Writing – Review & Editing, Project administration. **Alexander Chen**: Conceptualization, Methodology, Validation, Formal analysis, Investigation, Data curation, Visualization, Writing – Original draft preparation, Writing – Review & Editing, Project administration. **Van K. Ninh**.: Investigation, Formal Analysis, Data Collection, Writing – review & editing. **Maxwell C. McCabe**: Investigation, Formal Analysis, Data Collection, Writing – review & editing. **Quincy Lyons**: Investigation, Formal Analysis, Data Collection, Writing – review & editing. **Benjamin D. Bridgelal**: Investigation, Formal Analysis, Data Collection, Writing – review & editing. **Connor Uhre**: Investigation, Formal Analysis, Data Collection, Writing – review & editing. **Julian Cheng**: Investigation, Formal Analysis, Data Collection, Writing – review & editing. **Kate E. Reimold**: Investigation, Formal Analysis, Data Collection, Writing – review & editing. **Selena Cao**: Investigation, Formal Analysis, Data Collection, Writing – review & editing. **Kirk C. Hansen**: Resources, Supervision, Funding Acquisition. **Kevin R. King**: Resources, Supervision, Funding Acquisition. **Karen L. Christman**: Conceptualization, Validation, Resources, Writing – Review & Editing, Supervision, Funding Acquisition.

## Conflicts of Interest

KLC is co‐founder, consultant, board member of, and holds equity interest and receives income from Ventrix Bio Inc.

## Supporting information




**Supporting File 1**: advs78446‐sup‐0001‐SuppMat.docx.

**Supporting File 2**: advs78446‐sup‐0002‐SuppMat.xlsx.

## Data Availability

Single nucleus RNA sequencing and spatial transcriptomic sequencing datasets have been deposited to the Gene Expression Omnibus and are available under accession GSE299772 and GSE31076. All other data supporting the findings in this study are included in the main article and associated files.

## References

[1] S. S. Martin , A. W. Aday , N. B. Allen , et al., “2025 Heart Disease and Stroke Statistics: A Report of US and Global Data From the American Heart Association,” Circulation 151, no. 8 (2025): e41–e660, 10.1161/CIR.0000000000001303. PMC12256702 39866113

[2] N. G. Frangogiannis , “Pathophysiology of Myocardial Infarction,” in Comprehensive Physiology (John Wiley & Sons, 2015), 1841–1875. 10.1002/cphy.c150006 26426469

[3] N. G. Frangogiannis , C. W. Smith , and M. L. Entman , “The Inflammatory Response in Myocardial Infarction,” Cardiovascular Research 53, no. 1 (2002): 31–47, 10.1016/S0008-6363(01)00434-5. 11744011

[4] D. Gabriel‐Costa , “The Pathophysiology of Myocardial Infarction‐Induced Heart Failure,” Pathophysiology 25, no. 4 (2018): 277–284, 10.1016/j.pathophys.2018.04.003. 29685587

[5] M. Jessup and S. Brozena , “Heart Failure,” New England Journal of Medicine 348, no. 20 (2003): 2007–2018, 10.1056/NEJMra021498. 12748317

[6] C. L. Kerrigan and M. A. Stotland , “Ischemia Reperfusion Injury: A Review,” Microsurgery 14, no. 3 (1993): 165–175, 10.1002/micr.1920140307. 8479314

[7] B. Dorweiler , D. Pruefer , T. B. Andrasi , et al., “Ischemia‐Reperfusion Injury: Pathophysiology and Clinical Implications,” European Journal of Trauma and Emergency Surgery 33, no. 6 (2007): 600–612, 10.1007/s00068-007-7152-z. 26815087

[8] J. L. Ungerleider and K. L. Christman , “Concise Review: Injectable Biomaterials for the Treatment of Myocardial Infarction and Peripheral Artery Disease: Translational Challenges and Progress,” Stem Cells Translational Medicine 3, no. 9 (2014): 1090–1099, 10.5966/sctm.2014-0049. PMC4149304 25015641

[9] T. Liu , Y. Hao , Z. Zhang , et al., “Advanced Cardiac Patches for the Treatment of Myocardial Infarction,” Circulation 149, no. 25 (2024): 2002–2020, 10.1161/CIRCULATIONAHA.123.067097. PMC11191561 38885303

[10] J. H. Traverse , T. D. Henry , N. Dib , et al., “First‐in‐Man Study of a Cardiac Extracellular Matrix Hydrogel in Early and Late Myocardial Infarction Patients,” JACC: Basic to Translational Science 4, no. 6 (2019): 659–669, 10.1016/j.jacbts.2019.07.012. PMC6834965 31709316

[11] S. L. Suarez , A. A. Rane , A. Muñoz , et al., “Intramyocardial Injection of Hydrogel With High Interstitial Spread Does not Impact Action Potential Propagation,” Acta Biomaterialia 26 (2015): 13–22, 10.1016/j.actbio.2015.08.004. PMC4772723 26265060

[12] R. M. Wang , J. M. Mesfin , J. Hunter , et al., “Myocardial Matrix Hydrogel Acts as a Reactive Oxygen Species Scavenger and Supports a Proliferative Microenvironment for Cardiomyocytes,” Acta Biomaterialia 152 (2022): 47–59, 10.1016/j.actbio.2022.08.050. 36041648

[13] S. B. Seif‐Naraghi , J. M. Singelyn , M. A. Salvatore , et al., “Safety and Efficacy of an Injectable Extracellular Matrix Hydrogel for Treating Myocardial Infarction,” Science Translational Medicine 5, no. 173 (2013): 173ra25, 10.1126/scitranslmed.3005503. PMC3848875 23427245

[14] V. Menon , J. G. Webb , L. D. Hillis , et al., “Outcome and Profile of Ventricular Septal Rupture With Cardiogenic Shock After Myocardial Infarction: A Report From the SHOCK Trial Registry,” JACC 36 (2000): 1110–1116, 10.1016/S0735-1097(00)00878-0. 10985713

[15] Y. Birnbaum , M. C. Fishbein , C. Blanche , and R. J. Siegel , “Ventricular Septal Rupture After Acute Myocardial Infarction,” New England Journal of Medicine 347, no. 18 (2002): 1426–1432, 10.1056/NEJMra020228. 12409546

[16] M. Lin , J. Qin , S. Lin , et al., “Bioengineered Hydrogels for Restoring Cardiac Electronic Activity after Myocardial Infarction,” Advanced Healthcare Materials 14 (2025): 2502058, 10.1002/adhm.202502058. 40741668

[17] A. Chen , J. M. Mesfin , N. C. Gianneschi , and K. L. Christman , “Intravascularly Deliverable Biomaterial Platforms for Tissue Repair and Regeneration Post‐Myocardial Infarction,” Advanced Materials 36, no. 43 (2024): 2300603, 10.1002/adma.202300603. PMC10539487 36989469

[18] V. Z. Beachley , M. T. Wolf , K. Sadtler , et al., “Tissue Matrix Arrays for High‐Throughput Screening and Systems Analysis of Cell Function,” Nature Methods 12, no. 12 (2015): 1197–1204, 10.1038/nmeth.3619. PMC4666781 26480475

[19] M. T. Spang , R. Middleton , M. Diaz , et al., “Intravascularly Infused Extracellular Matrix as a Biomaterial for Targeting and Treating Inflamed Tissues,” Nature Biomedical Engineering 7, no. 2 (2023): 94–109, 10.1038/s41551-022-00964-5. PMC10166066 36581694

[20] M. L. Karkanitsa , R. M. Wang , A. C. Lyons , et al., “An Infusible Extracellular Matrix Biomaterial Improves Survival in a Model of Severe Systemic Inflammation,” npj Regen Med 10, no. 1 (2025): 48, 10.1038/s41536-025-00435-w. PMC12578793 41173831

[21] X. Li , J. Zou , Z. He , Y. Sun , X. Song , and W. He , “The Interaction Between Particles and Vascular Endothelium in Blood Flow,” Advanced Drug Delivery Reviews 207 (2024): 115216, 10.1016/j.addr.2024.115216. 38387770

[22] J. Nong , P. M. Glassman , and V. R. Muzykantov , “Targeting Vascular Inflammation Through Emerging Methods and Drug Carriers,” Advanced Drug Delivery Reviews 184 (2022): 114180, 10.1016/j.addr.2022.114180. PMC9035126 35271986

[23] E. Carboni , K. Tschudi , J. Nam , X. Lu , and A. W. K. Ma , “Particle Margination and Its Implications on Intravenous Anticancer Drug Delivery,” AAPS PharmSciTech [Electronic Resource] 15, no. 3 (2014): 762–771, 10.1208/s12249-014-0099-6. PMC4037490 24687242

[24] S. Muro , C. Garnacho , J. A. Champion , et al., “Control of Endothelial Targeting and Intracellular Delivery of Therapeutic Enzymes by Modulating the Size and Shape of ICAM‐1‐targeted Carriers,” Molecular Therapy 16, no. 8 (2008): 1450–1458, 10.1038/mt.2008.127. PMC2810502 18560419

[25] T. J. Moyer , H. A. Kassam , E. S. M. Bahnson , et al., “Shape‐Dependent Targeting of Injured Blood Vessels by Peptide Amphiphile Supramolecular Nanostructures,” Small 11, no. 23 (2015): 2750–2755, 10.1002/smll.201403429. PMC4478239 25649528

[26] J. L. Ungerleider , T. D. Johnson , N. Rao , and K. L. Christman , “Fabrication and Characterization of Injectable Hydrogels Derived From Decellularized Skeletal and Cardiac Muscle,” Methods 84 (2015): 53–59, 10.1016/j.ymeth.2015.03.024. PMC4526417 25843605

[27] M. D. Diaz , R. M. Kandell , J. R. Wu , A. Chen , K. L. Christman , and E. J. Kwon , “Infusible Extracellular Matrix Biomaterial Promotes Vascular Integrity and Modulates the Inflammatory Response in Acute Traumatic Brain Injury,” Adv Healthcare Materials 12, no. 25 (2023): 2300782, 10.1002/adhm.202300782. PMC10592293 37390094

[28] A. Chen , M. B. Nguyen , J. Cheng , et al., “Elucidating Mechanisms of Gelation, Fiber Assembly, and Stability of Injectable Decellularized Extracellular Matrix Biomaterials,” Biopolymers 116, no. 4 (2025): 70037, 10.1002/bip.70037. PMC12188951 40558083

[29] J. M. Mesfin , A. Chen , Q. P. Lyons , et al., “Infusible Extracellular Matrix Biomaterial Enhances Cell‐Specific Pro‐Repair Responses Following Acute Myocardial Infarction,” Adv Healthcare Materials 15 (2025): 03469, 10.1002/adhm.202503469. PMC13378492 41414646

[30] F. Li , W. Li , S. A. Johnson , D. A. Ingram , M. C. Yoder , and S. F. Badylak , “Low‐Molecular‐Weight Peptides Derived from Extracellular Matrix as Chemoattractants for Primary Endothelial Cells,” Endothelium 11, no. 3‐4 (2004): 199–206, 10.1080/10623320490512390. 15370297

[31] F. Moffatt , P. Senkans , and D. Ricketts , “Approaches Towards the Quantitative Analysis of Peptides and Proteins by Reversed‐Phase High‐Performance Liquid Chromatography in the Absence of a Pure Reference Sample,” Journal of Chromatography A 891, no. 2 (2000): 235–242, 10.1016/S0021-9673(00)00620-8. 11043783

[32] V. S. LeBleu , B. MacDonald , and R. Kalluri , “Structure and Function of Basement Membranes,” Experimental Biology and Medicine 232, no. 9 (2007): 1121–1129, 10.3181/0703-MR-72. 17895520

[33] E. Boland , F. Quondamatteo , and T. Van Agtmael , “The Role of Basement Membranes in Cardiac Biology and Disease,” Bioscience Reports 41, no. 8 (2021): BSR20204185, 10.1042/BSR20204185. PMC8390786 34382650

[34] D. J. S. Hulmes , “Collagen Diversity, Synthesis and Assembly,” in Collagen: Structure and Mechanics, ed.: P. Fratzl (Springer, 2008), 15–47.

[35] J. Nawata , “Differential Expression of a1, a3 and a5 Integrin Subunits in Acute and Chronic Stages of Myocardial Infarction in Rats,” Cardiovascular Research 19 (1999): 1551. 10.1016/s0008-6363(99)00117-0 10536667

[36] M. Sun , M. A. Opavsky , D. J. Stewart , et al., “Temporal Response and Localization of Integrins β1 and β3 in the Heart After Myocardial Infarction: Regulation by Cytokines,” Circulation 107, no. 7 (2003): 1046–1052, 10.1161/01.CIR.0000051363.86009.3C. 12600920

[37] S. H. Merchant , D. M. Gurule , and R. S. Larson , “Amelioration of Ischemia‐Reperfusion Injury With Cyclic Peptide Blockade of ICAM‐1,” American Journal of Physiology‐Heart and Circulatory Physiology 284, no. 4 (2003): H1260–H1268, 10.1152/ajpheart.00840.2002. 12595290

[38] J. L. Park and B. R. Lucchesi , “Mechanisms of Myocardial Reperfusion Injury,” The Annals of Thoracic Surgery 68 (1999): 1905–1912, 10.1016/S0003-4975(99)01073-5. 10585102

[39] Z. Wei , M. W. Rolle , and T. A. Camesano , “LL37 and Collagen‐Binding Domain‐Mediated LL37 Binding With Type I Collagen: Quantification via QCM‐D,” Colloids and Surfaces B: Biointerfaces 220 (2022): 112852, 10.1016/j.colsurfb.2022.112852. 36179608

[40] J. Malmström , H. Agheli , P. Kingshott , and D. S. Sutherland , “Viscoelastic Modeling of Highly Hydrated Laminin Layers at Homogeneous and Nanostructured Surfaces: Quantification of Protein Layer Properties Using QCM‐D and SPR,” Langmuir 23, no. 19 (2007): 9760–9768, 10.1021/la701233y. 17691829

[41] S. Eom , E. Yu , and S.‐J. Choi , “Functionalization of Quartz Crystal Microbalances With Liposomes Containing the N‐Hydroxysuccinimide Ester of Palmitic Acid,” Analytical Biochemistry 443, no. 1 (2013): 78–80, 10.1016/j.ab.2013.08.017. 23993905

[42] M. Gagliardi , L. Colagiorgio , and M. Cecchini , “A Fast and Reliable Method Based on QCM‐D Instrumentation for the Screening of Nanoparticle/Blood Protein Interactions,” Biosensors 13, no. 6 (2023): 607, 10.3390/bios13060607. PMC10296749 37366972

[43] T. Moazzenzade , L. Loohuis , S. G. Lemay , and J. Huskens , “Analyzing Binding Specificity in a Microparticle‐Based DNA Displacement Assay Using Multiharmonic QCM‐D,” Langmuir 40, no. 49 (2024): 26067–26075, 10.1021/acs.langmuir.4c03510. PMC11636201 39587401

[44] A. R. Patel , K. K. Kanazawa , and C. W. Frank , “Antibody Binding to a Tethered Vesicle Assembly Using QCM‐D,” Analytical Chemistry 81, no. 15 (2009): 6021–6029, 10.1021/ac802756v. 19580260

[45] M. Ohno , N. Ohno , and N. A. Kefalides , “Studies on Human Laminin and Laminin‐Collagen Complexes,” Connective Tissue Research 25, no. 3‐4 (1991): 251–262, 10.3109/03008209109029161. 2060301

[46] R. W. N. Nugroho , R. Harjumäki , X. Zhang , et al., “Quantifying the Interactions Between Biomimetic Biomaterials – Collagen I, Collagen IV, Laminin 521 and Cellulose Nanofibrils – by Colloidal Probe Microscopy,” Colloids and Surfaces B: Biointerfaces 173 (2019): 571–580, 10.1016/j.colsurfb.2018.09.073. 30347384

[47] X. Li , B. Redfors , M. Sáinz‐Jaspeado , et al., “Suppressed Vascular Leakage and Myocardial Edema Improve Outcome From Myocardial Infarction,” Frontiers in Physiology 11 (2020): 763, 10.3389/fphys.2020.00763. PMC7363985 32733273

[48] R. Garcia , C. Bouleti , M. Sirol , et al., “VEGF‐A Plasma Levels are Associated With Microvascular Obstruction in Patients With ST‐Segment Elevation Myocardial Infarction,” International Journal of Cardiology 291 (2019): 19–24, 10.1016/j.ijcard.2019.02.067. 30910283

[49] J. M. Mesfin , V. K. Ninh , M. D. Diaz , et al., “Uncovering the Regional and Cell Specific Bioactivity of Injectable Extracellular Matrix Biomaterials in Myocardial Infarction through Spatial and Single Nucleus Transcriptomics,” BioRxiv 2025 (2025): 661525. 10.1038/s41467-025-65351-5 PMC12644877 41285846

[50] V. K. Ninh , D. M. Calcagno , J. D. Yu , et al., “Spatially Clustered Type I Interferon Responses at Injury Borderzones,” Nature 633, no. 8028 (2024): 174–181, 10.1038/s41586-024-07806-1. PMC11374671 39198639

[51] M. Koyanagi , P. Bushoven , M. Iwasaki , C. Urbich , A. M. Zeiher , and S. Dimmeler , “Notch Signaling Contributes to the Expression of Cardiac Markers in Human Circulating Progenitor Cells,” Circulation Research 101, no. 11 (2007): 1139–1145, 10.1161/CIRCRESAHA.107.151381. 17967789

[52] C. Pellet‐Many , V. Mehta , L. Fields , et al., “Neuropilins 1 and 2 Mediate Neointimal Hyperplasia and Re‐Endothelialization Following Arterial Injury,” Cardiovascular Research 108, no. 2 (2015): 288–298, 10.1093/cvr/cvv229. PMC4614691 26410366

[53] D. Nichol and H. Stuhlmann , “EGFL7: A Unique Angiogenic Signaling Factor in Vascular Development and Disease,” Blood 119, no. 6 (2012): 1345–1352, 10.1182/blood-2011-10-322446. PMC3286203 22160377

[54] X. Liu , E. De la Cruz , X. Gu , et al., “Lymphoangiocrine Signals Promote Cardiac Growth and Repair,” Nature 588, no. 7839 (2020): 705–711, 10.1038/s41586-020-2998-x. PMC7770123 33299187

[55] X. Lin , W. Liu , Y. Chu , et al., “Activation of AHR by ITE Improves Cardiac Remodelling and Function in Rats After Myocardial Infarction,” ESC Heart Failure 10, no. 6 (2023): 3622–3636, 10.1002/ehf2.14532. PMC10682871 37798907

[56] J. M. Vieira , S. Norman , C. Villa del Campo , et al., “The Cardiac Lymphatic System Stimulates Resolution of Inflammation Following Myocardial Infarction,” Journal of Clinical Investigation 128, no. 8 (2018): 3402–3412, 10.1172/JCI97192. PMC6063482 29985167

[57] A. I. Nyka?nen , H. Sandelin , R. Krebs , et al., “Targeting Lymphatic Vessel Activation and CCL21 Production by Vascular Endothelial Growth Factor Receptor‐3 Inhibition Has Novel Immunomodulatory and Antiarteriosclerotic Effects in Cardiac Allografts,” Circulation 121, no. 12 (2010): 1413–1422, 10.1161/CIRCULATIONAHA.109.910703. 20231530

[58] Y. J. Kim , J. M. Yu , H. J. Joo , et al., “Role of CD9 in Proliferation and Proangiogenic Action of Human Adipose‐Derived Mesenchymal Stem Cells,” Pflugers Arch – Eur J Physiol 455, no. 2 (2007): 283–296, 10.1007/s00424-007-0285-4. 17668233

[59] M. Segarra , H. Ohnuki , D. Maric , et al., “Semaphorin 6A Regulates Angiogenesis by Modulating VEGF Signaling,” Blood 120, no. 19 (2012): 4104–4115, 10.1182/blood-2012-02-410076. PMC3496961 23007403

[60] D. Corà , E. Astanina , E. Giraudo , and F. Bussolino , “Semaphorins in Cardiovascular Medicine,” Trends in Molecular Medicine 20, no. 10 (2014): 589–598, 10.1016/j.molmed.2014.07.005. 25154329

[61] Y. Bai , L. Chen , F. Guo , et al., “EphrinB2‐mediated CDK5/ISL1 pathway enhances cardiac lymphangiogenesis and alleviates ischemic injury by resolving post‐MI inflammation,” Sig Transduct Target Ther 9, no. 1 (2024): 326, 10.1038/s41392-024-02019-4. PMC11574162 39557830

[62] Quancheng Lin , Jian Liu , Qingyang Chen , Yan Li , Feiyue Dai , and Xinyu Chen , “Mechanisms of Myocardial Ischemic Injury Repair by Bone Marrow Mesenchymal Stem Cell‐Derived miR‐183‐5P Targeting FOXO1,” Cellular and Molecular Biology 69, no. 4 (2023): 94–100, 10.14715/cmb/2023.69.4.14. 37329542

[63] R. Kalluri , “Basement Membranes: Structure, Assembly and Role in Tumour Angiogenesis,” Nature Reviews Cancer 3, no. 6 (2003): 422–433, 10.1038/nrc1094. 12778132

[64] R. Kalluri and D. Cosgrove , “Assembly of Type IV Collagen,” Journal of Biological Chemistry 275, no. 17 (2000): 12719–12724, 10.1074/jbc.275.17.12719. 10777566

[65] M. M. Gladka , A. Kohela , B. Molenaar , et al., “Cardiomyocytes Stimulate Angiogenesis After Ischemic Injury in a ZEB2‐Dependent Manner,” Nature Communications 12, no. 1 (2021): 84, 10.1038/s41467-020-20361-3. PMC7782784 33398012

[66] K. Ando , L. Tong , D. Peng , et al., “KCNJ8/ABCC9‐Containing K‐ATP Channel Modulates Brain Vascular Smooth Muscle Development and Neurovascular Coupling,” Developmental Cell 57, no. 11 (2022): 1383–1399, 10.1016/j.devcel.2022.04.019. 35588738

[67] V. Domenga , P. Fardoux , P. Lacombe , et al., “ *Notch3*is Required for Arterial Identity and Maturation of Vascular Smooth Muscle Cells,” Genes & development 18, no. 22 (2004): 2730–2735, 10.1101/gad.308904. PMC528893 15545631

[68] L. Segal , S. Etzion , S. Elyagon , et al., “Dock10 Regulates Cardiac Function under Neurohormonal Stress,” IJMS 23, no. 17 (2022): 9616, 10.3390/ijms23179616. PMC9455810 36077014

[69] S. Wang , S. Huang , S. Johnson , et al., “Tissue‐Specific Angiogenic and Invasive Properties of Human Neonatal Thymus and bone MSCs: Role of SLIT3‐ROBO1,” Stem Cells Translational Medicine 9, no. 9 (2020): 1102–1113, 10.1002/sctm.19-0448. PMC7445019 32470195

[70] L. Zhuang , X. Zong , Q. Yang , Q. Fan , and R. Tao , “Interleukin‐34‐NF‐κB Signaling Aggravates Myocardial Ischemic/Reperfusion Injury by Facilitating Macrophage Recruitment and Polarization,” eBioMedicine 95 (2023): 104744, 10.1016/j.ebiom.2023.104744. PMC10433018 37556943

[71] H. S. Fayed , M. Z. Bakleh , J. V. Ashraf , A. Howarth , D. Ebner , and A. Al Haj Zen , “Selective ROCK Inhibitor Enhances Blood Flow Recovery after Hindlimb Ischemia,” IJMS 24, no. 19 (2023): 14410, 10.3390/ijms241914410. PMC10572734 37833857

[72] Z. J. Greenberg , D. A. Monlish , R. L. Bartnett , et al., “The Tetraspanin CD53 Regulates Early B Cell Development by Promoting IL‐7R Signaling,” The Journal of Immunology 204, no. 1 (2020): 58–67, 10.4049/jimmunol.1900539. PMC6926393 31748347

[73] X. Jiang , M. Cao , J. Wu , et al., “Protections of Transcription Factor BACH2 and Natural Product Myricetin Against Pathological Cardiac Hypertrophy and Dysfunction,” Frontiers in Physiology 13 (2022): 971424, 10.3389/fphys.2022.971424. PMC9465486 36105283

[74] H. You , F. Chang , H. Chen , Y. Wang , and W. Han , “Exploring the Role of CBLB in Acute Myocardial Infarction: Transcriptomic, Microbiomic, and Metabolomic Analyses,” J Transl Med 22, no. 1 (2024): 654, 10.1186/s12967-024-05425-y. PMC11247792 39004726

[75] P. Huebener , T. Abou‐Khamis , P. Zymek , et al., “CD44 Is Critically Involved in Infarct Healing by Regulating the Inflammatory and Fibrotic Response,” The Journal of Immunology 180, no. 4 (2008): 2625–2633, 10.4049/jimmunol.180.4.2625. 18250474

[76] M. He , Y. Yu , S. Ning , J. Han , and Z. Guo , “Effects of STAT4 on Myocardial Ischemia‑Reperfusion Injury and the Underlying Mechanisms,” Molecular Medicine Reports 30, no. 5 (2024): 197, 10.3892/mmr.2024.13321. PMC11391519 39239743

[77] Z. Yin , Y. Zou , D. Wang , et al., “Regulation of the Tec Family of Non‐Receptor Tyrosine Kinases in Cardiovascular Disease,” Cell Death Discov 8, no. 1 (2022): 119, 10.1038/s41420-022-00927-4. PMC8927484 35296647

[78] Y. Hou , Y. Wang , K. Tang , et al., “CD226 Deficiency Attenuates Cardiac Early Pathological Remodeling and Dysfunction via Decreasing Inflammatory Macrophage Proportion and Macrophage Glycolysis in STZ‐Induced Diabetic Mice,” The FASEB Journal 37, no. 8 (2023): 23047, 10.1096/fj.202300424RR. 37392373

[79] C. P. Manta , T. Leibing , M. Friedrich , et al., “Targeting of Scavenger Receptors Stabilin‐1 and Stabilin‐2 Ameliorates Atherosclerosis by a Plasma Proteome Switch Mediating Monocyte/Macrophage Suppression,” Circulation 146, no. 23 (2022): 1783–1799, 10.1161/CIRCULATIONAHA.121.058615. 36325910

[80] Q. Zhu , S. Liao , Y. Zhou , R. Zhou , and M. Dong , "CD16^3+^/Dab^2+^ Macrophages Alleviate Cardiac Hypertrophy via Nrg_2_/ErbB_4_‐Mediated Mitochondrial Reprogramming," preprint, bioXriv, May 26, 2025, 10.1101/2025.05.22.655661.

[81] Z. Jiang , J. J. Qin , Y. Zhang , et al., “LILRB_4_ Deficiency Aggravates the Development of Atherosclerosis and Plaque Instability by Increasing the Macrophage Inflammatory Response via NF‐κB Signaling,” Clinical Science 131, no. 17 (2017): 2275–2288, 10.1042/cs20170198. 28743735

[82] J. Yang , M. Zhang , L. Liu , et al., “LILRB4 Exacerbates Myocardial Ischemia‐Reperfusion Injury by Promoting Inflammation and Pyroptosis,” Biochimica et Biophysica Acta (BBA) – Molecular Basis of Disease 1871, no. 7 (2025): 167959, 10.1016/j.bbadis.2025.167959. 40550372

[83] M. C. Louwe , B. Lammers , M. A. Frias , et al., “Abca1 Deficiency Protects the Heart Against Myocardial Infarction‐Induced Injury,” Atherosclerosis 251 (2016): 159–163, 10.1016/j.atherosclerosis.2016.06.023. 27323229

[84] N. Nishiyama , H. Koizumi , C. Nakahashi‐Oda , et al., “CD300a Immunoreceptor Regulates Ischemic Tissue Damage and Adverse Remodeling in the Mouse Heart and Kidney,” Journal of Clinical Investigation 135 (2025): 184984, 10.1172/JCI184984. PMC12483560 40705472

[85] J. Dai , J. Liu , Q. Zhang , et al., “Cathepsin C Is Involved in Macrophage M1 Polarization via p38/MAPK Pathway in Sudden Cardiac Death,” Cardiovascular Therapeutics 2021 (2021): 1–12, 10.1155/2021/6139732. PMC8536465 34737793

[86] J. M. Mesfin , A. Chen , Q. P. Lyons , et al., “Infusible Extracellular Matrix Biomaterial Enhances Cell‐Specific Pro‐Repair Responses Following Acute Myocardial Infarction,” bioRxiv 15 (2025): 03469. 10.1002/adhm.202503469 PMC13378492 41414646

[87] J. L. Dziki , D. S. Wang , C. Pineda , B. M. Sicari , T. Rausch , and S. F. Badylak , “Solubilized Extracellular Matrix Bioscaffolds Derived From Diverse Source Tissues Differentially Influence Macrophage Phenotype,” Journal of Biomedical Materials Research Part A 105, no. 1 (2017): 138–147, 10.1002/jbm.a.35894. 27601305

[88] P. F. Slivka , C. L. Dearth , T. J. Keane , et al., “Fractionation of an ECM Hydrogel Into Structural and Soluble Components Reveals Distinctive Roles in Regulating Macrophage Behavior,” Biomaterials Science 2, no. 10 (2014): 1521–1534, 10.1039/C4BM00189C. 26829566

[89] X. Xu , P. Zhang , D. Kwak , et al., “Cardiomyocyte Dimethylarginine Dimethylaminohydrolase‐1 (DDAH1) Plays an Important Role in Attenuating Ventricular Hypertrophy and Dysfunction,” Basic Research in Cardiology 112, no. 5 (2017): 55, 10.1007/s00395-017-0644-z. PMC6502637 28819685

[90] P. Ortiz‐Sánchez , M. Villalba‐Orero , M. M. López‐Olañeta , et al., “Loss of SRSF3 in Cardiomyocytes Leads to Decapping of Contraction‐Related mRNAs and Severe Systolic Dysfunction,” Circulation Research 125, no. 2 (2019): 170–183, 10.1161/CIRCRESAHA.118.314515. PMC6615931 31145021

[91] H. Cheng and W. Yan , “MiR‐433 Regulates Myocardial Ischemia Reperfusion Injury by Targeting NDRG4 Via the PI3K/Akt Pathway,” Shock 54, no. 6 (2020): 802–809, 10.1097/SHK.0000000000001532. 32187107

[92] M. Yan , J. Gao , M. Lan , et al., “DEAD‐box Helicase 17 (DDX17) Protects Cardiac Function by Promoting Mitochondrial Homeostasis in Heart Failure,” Sig Transduct Target Ther 9, no. 1 (2024): 127, 10.1038/s41392-024-01831-2. PMC11116421 38782919

[93] L. Yuan , S. Bu , M. Du , et al., “RNF207 Exacerbates Pathological Cardiac Hypertrophy via Post‐Translational Modification of TAB1,” Cardiovascular Research 119, no. 1 (2023): 183–194, 10.1093/cvr/cvac039. 35352799

[94] X. Liang , Y. Ding , Y. Zhang , et al., “Activation of NRG1‐ERBB4 Signaling Potentiates Mesenchymal Stem Cell‐Mediated Myocardial Repairs Following Myocardial Infarction,” Cell Death & Disease 6, no. 5 (2015): e1765–e1765, 10.1038/cddis.2015.91. PMC4669719 25996292

[95] R. D. Sullivan , A. K. Houng , I. P. Gladysheva , et al., “Corin Overexpression Reduces Myocardial Infarct Size and Modulates Cardiomyocyte Apoptotic Cell Death,” IJMS 21, no. 10 (2020): 3456, 10.3390/ijms21103456. PMC7278931 32422879

[96] F.‐L. Xiang , M. Guo , and K. E. Yutzey , “Overexpression of Tbx20 in Adult Cardiomyocytes Promotes Proliferation and Improves Cardiac Function After Myocardial Infarction,” Circulation 133, no. 11 (2016): 1081–1092, 10.1161/CIRCULATIONAHA.115.019357. PMC4792775 26841808

[97] B. Ding , J. I. Abe , H. Wei , et al., “Functional Role of Phosphodiesterase 3 in Cardiomyocyte Apoptosis: Implication in Heart Failure,” Circulation 111, no. 19 (2005): 2469–2476, 10.1161/01.CIR.0000165128.39715.87. PMC4108189 15867171

[98] N. Polidovitch , S. Yang , H. Sun , et al., “Phosphodiesterase Type 3A (PDE3A), but not Type 3B (PDE3B), Contributes to the Adverse Cardiac Remodeling Induced by Pressure Overload,” Journal of Molecular and Cellular Cardiology 132 (2019): 60–70, 10.1016/j.yjmcc.2019.04.028. 31051182

[99] Y. Wang , Y. Cao , S. Yamada , et al., “Cardiomyopathy and Worsened Ischemic Heart Failure in SM22‐α Cre‐Mediated Neuropilin‐1 Null Mice: Dysregulation of PGC1α and Mitochondrial Homeostasis,” ATVB 35, no. 6 (2015): 1401–1412, 10.1161/ATVBAHA.115.305566. PMC4441604 25882068

[100] J. Sun , C. Niu , W. Ye , et al., “FGF13 Is a Novel Regulator of NF‐κB and Potentiates Pathological Cardiac Hypertrophy,” iScience 23, no. 10 (2020): 101627, 10.1016/j.isci.2020.101627. PMC7567043 33089113

[101] A. Liu , Y. Zhang , S. Xun , G. Zhou , L. Lin , and Y. Mei , “Fibroblast Growth Factor 12 Attenuated Cardiac Remodeling via Suppressing Oxidative Stress,” Peptides 153 (2022): 170786, 10.1016/j.peptides.2022.170786. 35304156

[102] F. B. Engel , P. C. H. Hsieh , R. T. Lee , and M. T. Keating , “FGF1/p38 MAP Kinase Inhibitor Therapy Induces Cardiomyocyte Mitosis, Reduces Scarring, and Rescues Function After Myocardial Infarction,” Proceedings of the National Academy of Sciences of the United States of America 103, no. 42 (2006): 15546–15551, 10.1073/pnas.0607382103. PMC1622860 17032753

[103] X. Zhang , B. Tian , X. Cong , and Z. Ning , “SLIT3 Promotes Cardiac Fibrosis and Differentiation of Cardiac Fibroblasts by RhoA/ROCK1 Signaling Pathway,” Iranian Journal of Basic Medical Sciences 27, no. 7 (2024): 832–840, 10.22038/ijbms.2024.73812.16044. PMC11127076 38800023

[104] Y. Wang , D. Hua , K. Zhao , et al., “LTBP2 Knockdown Ameliorates Cardiac Fibrosis and Apoptosis via Attenuating NF‐κB Signaling Pathway and Oxidative Stress in Mice with Cardiac Hypertrophy,” Advanced Therapeutics 7, no. 8 (2024): 2300451, 10.1002/adtp.202300451.

[105] A. Heinen , R. Nederlof , P. Panjwani , et al., “IGF1 Treatment Improves Cardiac Remodeling after Infarction by Targeting Myeloid Cells,” Molecular Therapy 27, no. 1 (2019): 46–58, 10.1016/j.ymthe.2018.10.020. PMC6319026 30528085

[106] G. Sufen , Y. Xianghong , C. Yongxia , and P. Qian , “bFGF and PDGF‐BB Have a Synergistic Effect on the Proliferation, Migration and VEGF Release of Endothelial Progenitor Cells,” Cell Biology International 35, no. 5 (2011): 545–551, 10.1042/CBI20100401. 20961291

[107] K. Kalra , J. Eberhard , N. Farbehi , J. J. Chong , and M. Xaymardan , “Role of PDGF‐A/B Ligands in Cardiac Repair After Myocardial Infarction,” Frontiers in Cell and Developmental Biology 9 (2021): 669188, 10.3389/fcell.2021.669188. PMC8424099 34513823

[108] B. Fan , L. Ma , Q. Li , et al., “Role of PDGFs/PDGFRs Signaling Pathway in Myocardial Fibrosis of DOCA/Salt Hypertensive Rats,” American Journal of Translational Research 7 (2014): 16–27. PMC3885456 24427322

[109] X. Li , S. Zheng , W. Tan , et al., “Slit2 Protects Hearts Against Ischemia‐Reperfusion Injury by Inhibiting Inflammatory Responses and Maintaining Myofilament Contractile Properties,” Frontiers in Physiology 11 (2020): 228, 10.3389/fphys.2020.00228. PMC7135862 32292352

[110] W. G. Thomas , Y. Brandenburger , D. J. Autelitano , T. Pham , H. Qian , and R. D. Hannan , “Adenoviral‐Directed Expression of the Type 1A Angiotensin Receptor Promotes Cardiomyocyte Hypertrophy via Transactivation of the Epidermal Growth Factor Receptor,” Circulation Research 90, no. 2 (2002): 135–142, 10.1161/hh0202.104109. 11834705

[111] Y. Nakamura , S. Kita , Y. Tanaka , et al., “A Disintegrin and Metalloproteinase 12 Prevents Heart Failure by Regulating Cardiac Hypertrophy and Fibrosis,” American Journal of Physiology‐Heart and Circulatory Physiology 318, no. 2 (2020): H238–H251, 10.1152/ajpheart.00496.2019. 31774689

[112] X. Chen , X. Li , X. Wu , et al., “Integrin Beta‐Like 1 Mediates Fibroblast–Cardiomyocyte Crosstalk to Promote Cardiac Fibrosis and Hypertrophy,” Cardiovascular Research 119, no. 10 (2023): 1928–1941, 10.1093/cvr/cvad104. 37395147

[113] F. Bouzeghrane , D. P. Reinhardt , T. L. Reudelhuber , and G. Thibault , “Enhanced Expression of Fibrillin‐1, a Constituent of the Myocardial Extracellular Matrix in Fibrosis,” American Journal of Physiology‐Heart and Circulatory Physiology 289, no. 3 (2005): H982–H991, 10.1152/ajpheart.00151.2005. 15849235

[114] T. Zhao , W. Zhao , W. Meng , et al., “Vascular Endothelial Growth Factor‐D Mediates Fibrogenic Response in Myofibroblasts,” Molecular and Cellular Biochemistry 413, no. 1‐2 (2016): 127–135, 10.1007/s11010-015-2646-1. 26724950

[115] J. Barallobre‐Barreiro , T. Radovits , M. Fava , et al., “Extracellular Matrix in Heart Failure: Role of ADAMTS5 in Proteoglycan Remodeling,” Circulation 144, no. 25 (2021): 2021–2034, 10.1161/CIRCULATIONAHA.121.055732. PMC8687617 34806902

[116] L. Dyer , Y. Wu , M. Moser , and C. Patterson , “BMPER‐Induced BMP Signaling Promotes Coronary Artery Remodeling,” Developmental Biology 386, no. 2 (2014): 385–394, 10.1016/j.ydbio.2013.12.019. PMC4112092 24373957

[117] S. Ock , W. Ham , C. W. Kang , H. Kang , W. S. Lee , and J. Kim , “IGF‐1 Protects Against Angiotensin II‐Induced Cardiac Fibrosis by Targeting αSMA,” Cell Death & Disease 12, no. 7 (2021): 688, 10.1038/s41419-021-03965-5. PMC8270920 34244467

[118] E. P. Carter , K. K. Yoneten , N. Gavara , et al., “Opposing Roles for <Span Style = ”Font‐Variant:Small‐Caps;“>ADAMTS2</span>and <span style = ”font‐variant:Small‐caps;“>ADAMTS14</span>in Myofibroblast Differentiation and Function,” The Journal of Pathology 262, no. 1 (2024): 90–104, 10.1002/path.6214. PMC10953099 37929635

[119] Y. Wu , S. Chen , G. Huang , et al., “Transcriptome Analysis Reveals EBF1 Ablation‐Induced Injuries in Cardiac System,” Theranostics 14, no. 12 (2024): 4894–4915, 10.7150/thno.92060. PMC11373621 39239522

[120] S. Zhou , L. S. Chen , Y. Miyauchi , et al., “Mechanisms of Cardiac Nerve Sprouting After Myocardial Infarction in Dogs,” Circulation Research 95, no. 1 (2004): 76–83, 10.1161/01.RES.0000133678.22968.e3. 15166093

[121] M. Shimamura , H. Hayashi , N. Ju , et al., “R‐spondin 3/LGR4 (Leucine‐Rich Repeat‐Containing G Protein‐Coupled Receptor 4) Axis Is a Novel Inflammatory and Neurite Outgrowth Signaling System in the Ischemic Brain in Mice,” Stroke; A Journal of Cerebral Circulation 54, no. 6 (2023): 1606–1615, 10.1161/STROKEAHA.122.041970. 37165865

[122] X.‐Q. Peng , Y. Z. Li , C. Gu , et al., “Marcks Overexpression in Retinal Ganglion Cells Promotes Optic Nerve Regeneration,” Cell Death & Disease 15, no. 12 (2024): 906, 10.1038/s41419-024-07281-6. PMC11655864 39695101

[123] Q. Zhu , X. Lu , M. Chen , et al., “IGFBP5 Affects Cardiomyocyte Survival and Functional Recovery In Mice Following Myocardial Ischemia,” Communications Biology 7, no. 1 (2024): 1594, 10.1038/s42003-024-07304-0. PMC11607409 39613849

[124] A. Sreepada , M. Tiwari , and K. Pal , “Adhesion G Protein‐Coupled Receptor Gluing Action Guides Tissue Development and Disease,” Journal of Molecular Medicine 100, no. 10 (2022): 1355–1372, 10.1007/s00109-022-02240-0. 35969283

[125] S. M. McCurdy , Q. Dai , J. Zhang , et al., “SPARC Mediates Early Extracellular Matrix Remodeling Following Myocardial Infarction,” American Journal of Physiology 301 (2011): H1515–H1529. 10.1152/ajpheart.01070.2010 PMC3154667 21602472

[126] J.‐R. Lee , “Protein Tyrosine Phosphatase PTPRT as a Regulator of Synaptic Formation and Neuronal Development,” BMB Reports 48, no. 5 (2015): 249–255, 10.5483/BMBRep.2015.48.5.037. PMC4578563 25748173

[127] I. Barbosa , “Improved and Simple Micro Assay for Sulfated Glycosaminoglycans Quantification in Biological Extracts and its use in Skin and Muscle Tissue Studies,” Glycobiology 13, no. 9 (2003): 647–653, 10.1093/glycob/cwg082. 12773478

[128] D. Mellacheruvu , Z. Wright , A. L. Couzens , et al., “The CRAPome: A Contaminant Repository for Affinity Purification–Mass Spectrometry Data,” Nature Methods 10, no. 8 (2013): 730–736, 10.1038/nmeth.2557. PMC3773500 23921808

[129] A. T. Kong , F. V. Leprevost , D. M. Avtonomov , D. Mellacheruvu , and A. I. Nesvizhskii , “MSFragger: Ultrafast and Comprehensive Peptide Identification in Mass Spectrometry–Based Proteomics,” Nature Methods 14, no. 5 (2017): 513–520, 10.1038/nmeth.4256. PMC5409104 28394336

[130] D. M. Calcagno , N. Taghdiri , V. K. Ninh , et al., “Single‐Cell and Spatial Transcriptomics of the Infarcted Heart Define the Dynamic Onset of the Border Zone in Response to Mechanical Destabilization,” Nat Cardiovasc Res 1, no. 11 (2022): 1039–1055, 10.1038/s44161-022-00160-3. PMC11290420 39086770

[131] R. Satija , J. A. Farrell , D. Gennert , A. F. Schier , and A. Regev , “Spatial Reconstruction of Single‐Cell Gene Expression Data,” Nature Biotechnology 33, no. 5 (2015): 495–502, 10.1038/nbt.3192. PMC4430369 25867923

[132] A. Butler , P. Hoffman , P. Smibert , E. Papalexi , and R. Satija , “Integrating Single‐Cell Transcriptomic Data Across Different Conditions, Technologies, and Species,” Nature Biotechnology 36, no. 5 (2018): 411–420, 10.1038/nbt.4096. PMC6700744 29608179

[133] T. Stuart , A. Butler , P. Hoffman , et al., “Comprehensive Integration of Single‐Cell Data,” Cell 177, no. 7 (2019): 1888–1902.e21, 10.1016/j.cell.2019.05.031. PMC6687398 31178118

[134] Y. Hao , S. Hao , E. Andersen‐Nissen , et al., “Integrated Analysis of Multimodal Single‐Cell Data,” Cell 184, no. 13 (2021): 3573–3587.e29, 10.1016/j.cell.2021.04.048. PMC8238499 34062119

[135] Y. Hao , T. Stuart , M. H. Kowalski , et al., “Dictionary Learning for Integrative, Multimodal and Scalable Single‐Cell Analysis,” Nature Biotechnology 42, no. 2 (2024): 293–304, 10.1038/s41587-023-01767-y. PMC10928517 37231261

