# Supplementary material for: An Infusible Decellularized Extracellular Matrix Material Binds to Vasculature in Infarcted Myocardium and Induces Pro‐Reparative Gene Expression Following Acute Myocardial Infarction Through Inherent Avidity and Bioactive Signaling
Source: Adv Sci (Weinh). 2026 Oct 29:e78446. Online ahead of print. doi: 10.1002/advs.78446 (PMC13720270; doi:10.1002/advs.78446)
Supplement: Supplementary file 1 [file ADVS-9999-e78446-s001.docx]

**Supporting Information:**
**An infusible decellularized extracellular matrix material binds to vasculature in infarcted myocardium and induces pro-reparative gene expression following acute myocardial infarction through inherent avidity and bioactive signaling**

Michael B. Nguyen ^1,2^, Alexander Chen ^2,3^, Quincy Lyons ^1,2^, Van K. Ninh ^5^, Maxwell C. McCabe ^6^, Colin Luo ^1,2^, Benjamin D. Bridgelal ^1,2^, Connor Uhre ^1,2^, Julian Cheng ^1,2^, Kate E. Reimold ^1,2^, Selena Cao ^1,2^, Kirk C. Hansen ^6^, Kevin R. King ^1,5^, and Karen L. Christman *^1,2,3,4^

1. Shu Chien-Gene Lay Department of Bioengineering, University of California San Diego

2. Sanford Consortium for Regenerative Medicine, University of California San Diego

3. Program in Materials Science and Engineering, University of California San Diego

4. Sanford Stem Cell Institute, University of California San Diego

5. Department of Medicine, Division of Cardiology, University of California San Diego

6. Department of Biochemistry and Molecular Genetics, School of Medicine, University of Colorado, Aurora, Colorado 80045, USA

*Corresponding author: [kchristman@ucsd.edu](mailto:kchristman@ucsd.edu)

**Figure S1. iECM quality control metrics and fractionation.** a) Quantified residual dsDNA within the iECM and its components showing adequate decellularization and b) residual SDS showing sufficient removal of the detergent from after decellularization. c) Image of solubility-based fractionation of iECM in 50% ethanol showing solubilized low MW (red) and pelleted insoluble high MW (green). N = 3 technical replicates

**Figure S2. Assay verification data and interactions between the high MW vs low MW.** The 3^rd^ overtone adjusted frequency shift associated with a) flowing an antibody over the surface of the chemically conjugated ECM proteins and cell surface receptor proteins and b) when one component was conjugated and the other flowed. Third overtone frequency shifts for binding of laminin 111 and type I collagen antibodies were similar to previous reports of antibody binding to ECM coatings via QCM with n = 3 technical replicates per antibody and flow procedure.

Figure S3. iECM and components retention and conjugation efficiency. a) Near infrared imaging of transverse heart sections demonstrating left ventricular localization of complete iECM and iECM fractions. The b) Fluorescent images of high MW components vs saline with adjusted dynamic ranges. c) Conjugation efficiency of the fluorophore for high and low MW components. Statistical significance determined with a Welch’s two-tailed unpaired t-test, **** < 0.0001.

**Figure S4. SN sample QC prior to nuclei isolation and sequencing preparation.** (a-b) Representative H&E images of infarct quantification strategy displaying the three ROIs used for infarct percentage quantification and exclusion. c) Bar graph showing the distribution of quantified infarcts included for each treatment with n = 5-6 biological replicates per treatment.

 **Figure S5. Single Nucleus RNA Sequencing Quality Control and Cell Type Overview.** a-b) Quality metrics of samples and replicates for single nucleus RNA sequencing samples via features per nuclei (nFeatures) and genes per sample (nCounts). Significant ambient RNA contamination existed within the low MW replicates therefore reducing the features. However, the counts for the low MW replicates were similar suggesting a preservation of the gene expression signal after ambient RNA cleaning. c) The relative percentages of cell for each cell type identified per treatment and the d) associated UMAPs. Sample size n=8 animals pooled into n=4 sequencing replicates for iECM, n=6 animals pooled into n=3 sequencing replicates for saline and n=4 animals pooled into n=2 sequencing replicates for high MW and low MW. Total cells captured: iECM = 151933 cells, low MW = 146616 cells, high MW = 66734 cells, and saline = 125954 cells.

**Figure S6**. **Breakdown of endothelial cell subpopulations with feature plots of representative genes.** The subpopulations of endothelial cells for a) iECM vs saline, b) high MW vs saline and c) low MW vs saline.

**Figure S7**. **Breakdown of mural cell subpopulations with feature plots of representative genes.** The subpopulations of endothelial cells for a) iECM vs saline, b) high MW vs saline and c) low MW vs saline.

**Figure S8**. **Breakdown of B and dendritic cell subpopulations with feature plots of representative genes.** The subpopulations of endothelial cells for a) iECM vs saline, b) high MW vs saline and c) low MW vs saline.

**Figure S9**. **Breakdown of T and NK cell subpopulations with feature plots of representative genes.** The subpopulations of endothelial cells for a) iECM vs saline, b) high MW vs saline and c) low MW vs saline.

**Figure S10**. **Breakdown of macrophage subpopulations with feature plots of representative genes.** The subpopulations of endothelial cells for a) iECM vs saline, b) high MW vs saline and c) low MW vs saline.

**Figure S11.** **Breakdown of cardiomyocytes subpopulations with feature plots of representative genes.** The subpopulations of endothelial cells for a) iECM vs saline, b) high MW vs saline and c) low MW vs saline.

 **Figure S12**. **Breakdown of fibroblast subpopulations with feature plots of representative genes.** The subpopulations of endothelial cells for a) iECM vs saline, b) high MW vs saline and c) low MW vs saline.

**Supplementary Tables:**

**SI Table 1: Raw spectral intensity of matched proteins from mass spectrometry based proteomic analysis of iECM high MW and low MW batches**

**SI Table 2: Raw spectral intensity of matched peptide sequences from mass spectrometry based proteomic analysis of iECM high MW and low MW batches**

**SI Table 3: Quality control data for snRNAseq analysis showing detected number of cells types for iECM, high MW iECM, low MW iECM, and saline treated animals**

**SI Table 4: Differentially expressed genes for endothelial cells showing comparisons between iECM, high MW iECM, or low MW iECM compared to saline**

**SI Table 5: Differentially expressed genes for lymphatic endothelial cells showing comparisons between iECM, high MW iECM, or low MW iECM compared to saline**

**SI Table 6: Differentially expressed genes for mural cells showing comparisons between iECM, high MW iECM, or low MW iECM compared to saline**

**SI Table 7: Differentially expressed genes for B cells showing comparisons between iECM, high MW iECM, or low MW iECM compared to saline**

**SI Table 8: Differentially expressed genes for T cells showing comparisons between iECM, high MW iECM, or low MW iECM compared to saline**

**SI Table 9: Differentially expressed genes for macrophages showing comparisons between iECM, high MW iECM, or low MW iECM compared to saline**

**SI Table 10: Differentially expressed genes for fibroblasts showing comparisons between iECM, high MW iECM, or low MW iECM compared to saline**

**SI Table 11: Differentially expressed genes for cardiomyocytes showing comparisons between iECM, high MW iECM, or low MW iECM compared to saline**

**SI Table 12: Differentially expressed genes for neuronal showing comparisons between iECM, high MW iECM, or low MW iECM compared to saline**
